# A new extended Chen distribution for modelling COVID-19 data

**DOI:** 10.1371/journal.pone.0316235

**Published:** 2025-01-03

**Authors:** Amani S. Alghamdi, Lulah Alnaji

**Affiliations:** 1 Department of Statistics, Faculty of Science, King Abdulaziz University, Jeddah, Saudi Arabia; 2 Department of Mathematics, College of Science, University of Hafr Al Batin, Hafar Al-Batin, Saudi Arabia; Covenant University, NIGERIA

## Abstract

In this paper, we propose a new flexible statistical distribution, the Topp-Leone Exponentiated Chen distribution, to model real-world data effectively, with a particular focus on COVID-19 data. The motivation behind this study is the need for a more flexible distribution that can capture various hazard rate shapes (e.g., increasing, decreasing, bathtub) and provide better fitting performance compared to existing models such as the Chen and exponentiated Chen distributions. The principal results include the derivation of key statistical properties such as the probability density function, cumulative distribution function, moments, hazard rate function, and order statistics. Maximum likelihood estimation is employed to estimate the parameters of the TLEC distribution, and simulation studies demonstrate the efficiency of the maximum likelihood method. The innovation of this work is further validated by applying the TLEC distribution to real COVID-19 data, where it outperforms several related models. The study concludes with significant insights into how the TLEC distribution provides a more accurate and flexible approach to modeling real-world phenomena.

## 1 Introduction

In various contexts, lifetime data is crucial, and many applications, including those in biology, public health, science, administration, and engineering, heavily rely on lifetime data. Statistical distributions are used to simulate an object’s life and examine its key characteristics. When information is distributed properly, it can offer valuable insights that lead to wise conclusions and choices. There is always a need for new models that are more flexible, and as a consequence, many researchers are on the verge of adopting the new distribution with greater generality when the demand for more flexible distributions arises. Comprehensive knowledge of numerous techniques for creating families of continuous univariate distributions has been summarized in the review of [1]. One of the continuous distributions that is desirable as a generator is the Topp-Leone (TL) distribution. It was created by [2] to study the empirical data that has J-shaped histogram. Thus far, new generalized models based on the TL distribution are being developed as a result of several studies for use in real world applications. [3] derived the generalized Topp-Leone generated (GTL-G) family of distributions by including an additional parameter to the Topp-Leone generator of distributions for flexibility. A new family was introduced by [4] based on a variable of Topp-Leone random variable that is flexible in fitting different types of data. [5] studied the Topp Leone Gompertz distribution and studied its properties with applications. [6] suggested an expansion of the odd Fréchet family using the Topp-Leone method by including a shape parameter with the objective of increasing its overall flexibility. Based on complete and right censoring, [7] studied the properties of the Topp-Leone inverse Lomax distribution. [8] constructed a new family by applying T-X method to the inverted Topp-Leone distribution and studied its submodels in details under complete and censored samples. The quadratic rank transmutation map is used by [9] to suggest a novel extension of the power inverted Topp–Leone distribution and acceptance sampling plan was applied. [10] developed a new asymmetric model using Topp-Leone distribution as a generator and applied different estimation methods under progressive type-II censoring. [11] introduced a new generalized distribution of the inverted Topp-Leone distribution using the Kumaraswamy Marshall-Olkin family and applied the new model to real engineering datasets.

In particular, [12] discussed a Discrete Exponentiated-Chen (DEC) model and its applications, which extends the Chen distribution to suit discrete data scenarios. This further emphasizes the importance of developing new flexible models to fit various types of data, especially in reliability and lifespan data modeling. The DEC model introduces critical flexibility in hazard rate and probability mass function (PMF) shapes, similar to what is achieved by our proposed model. Such generalizations are essential for accurately modeling data across different domains, demonstrating the growing relevance of new statistical distributions like the one proposed in this paper.

According to [13], let *V* be a random variable of the TL distribution with the shape parameter *α*. Then, its CDF is defined as

$$\begin{array}{c} F_{TL}\left(v\right)=v^{\alpha }{\left(2-v\right)}^{\alpha },\;0<v<1,\alpha >0. \end{array}$$ (1)

The corresponding PDF is

$$\begin{array}{c} f_{TL}\left(v\right)=2\alpha v^{\alpha -1}\left(1-v\right){\left(2-v\right)}^{\alpha -1},\;0<v<1,\alpha >0. \end{array}$$ (2)

In survival analysis, it is fairly frequent to search for novel distributions with a lot of flexibility in the hazard rate function (HRF). Chen distribution was demonstrated by [14] that provides different shapes of the HRF such as bathtub and increasing failure rates. Let *X* be a non-negative random variable having Chen distribution. Then, its cumulative distribution function (CDF), denoted by *F*_*C*_(*x*; λ, *β*), and its PDF, denoted by *f*_*C*_(*x*; λ, *β*), respectively, given by

$$\begin{array}{c} F_{C}\left(x;\lambda ,\beta \right)=1-e^{\lambda (1-e^{x^{\beta }})},\;x>0,\lambda >0,\beta >0, \end{array}$$ (3)

and

$$\begin{array}{c} f_{C}\left(x;\lambda ,\beta \right)=\lambda \beta x^{\beta -1}e^{x^{\beta }+\lambda \left(1-e^{x^{\beta }}\right)},\;x>0,\lambda >0,\beta >0, \end{array}$$ (4)

where, λ is a scale parameter and *β* is a shape parameter. Numerous extensions in the literature have lately been made to the Chen distribution by [15–18]. Another method of extending Chen distribution is exponentiated Chen distribution which is considered as one of the most notable distributions to offer excellent flexibility in statistical modeling. It was introduced by [19] and studied by [20] by adding a shape parameter, *b* > 0, to the CDF of the Chen distribution as follows.

$$\begin{array}{c} \begin{array}{cc} G(x;\lambda ,\beta ,b) & ={\left[F_{C}\left(x;\lambda ,\beta \right)\right]}^{b}\\ & ={\left[1-e^{\lambda (1-e^{x^{\beta }})}\right]}^{b},\;x>0,\lambda >0,\beta >0,b>0. \end{array} \end{array}$$ (5)

The corresponding PDF is given by

$$\begin{array}{c} g\left(x;\lambda ,\beta ,b\right)=b\beta \lambda x^{\beta -1}e^{x^{\beta }}e^{\lambda (1-e^{x^{\beta }})}{\left(1-e^{\lambda (1-e^{x^{\beta }})}\right)}^{b-1}. \end{array}$$ (6)

In this paper, we will study an extension of the exponentiated Chen distribution using the TL distribution in Eq (1). The following are the main subjects covered in this article. In Section 2, the primary definition of the Topp-Leone exponentiated Chen (TLEC) distribution is addressed with the derivation of its alternative expression. In section 4, statistical properties for the new distribution are obtained. The maximum likelihood method for parameter estimation of the TLEC distribution is discussed in section 5. In section 6, a simulation study is conducted to assess the precision of the maximum likelihood estimate (MLE) of the distribution parameters. The performance of the suggested distribution on an actual data set is investigated in section 7. The discussion of the results is presented in. Finally, concluding remark is stated in section 9.

## 2 The Topp-Leone exponentiated Chen distribution

The TLEC distribution is presented in this section applying the concept of the TL-G family of distributions that was introduced by [21]. According to [5], if *X* is a continuous random variable with the CDF G(x) of the base distribution, then the CDF of the TL-G family of distribution is defined as follows:

$$\begin{array}{c} \begin{array}{cc} F{\left(x;\alpha \right)}_{TL-G} & ={\left[G\left(x\right)\right]}^{\alpha }{\left[2-G\left(x\right)\right]}^{\alpha }\\ & ={\left[1-{\left(\bar{G}\left(x\right)\right)}^{2}\right]}^{\alpha },\;x>0,\alpha >0. \end{array} \end{array}$$ (7)

By taking the derivative of Eq (7), the corresponding PDF is given by

$$\begin{array}{c} \begin{array}{cc} f{\left(x;\alpha \right)}_{TL-G} & =2\alpha g\left(x\right)\bar{G}\left(x\right){\left[G\left(x\right)\right]}^{\alpha -1}{\left[2-G\left(x\right)\right]}^{\alpha -1}\\ & =2\alpha g\left(x\right)\bar{G}\left(x\right){\left[1-{\left(\bar{G}\left(x\right)\right)}^{2}\right]}^{\alpha -1}, \end{array} \end{array}$$ (8)

where *α* is the shape parameter and $\bar{G}\left(x\right)=1-G\left(x\right)$.

The CDF of TLEC distribution is obtained by substituting Eq (5) in Eq (7) which is expressed as follows.

$$\begin{array}{c} \begin{array}{cc} F(x;\alpha ,\beta ,\lambda ,b) & ={\left[{\left(1-e^{\lambda (1-e^{x^{\beta }})}\right)}^{b}\right]}^{\alpha }{\left[2-{\left(1-e^{\lambda (1-e^{x^{\beta }})}\right)}^{b}\right]}^{\alpha }\\ & ={\left[1-{\left(1-\left({\left(1-e^{\lambda (1-e^{x^{\beta }})}\right)}^{b}\right)\right)}^{2}\right]}^{\alpha },\;x>0,\alpha ,\lambda ,\beta ,b>0\cdot \end{array} \end{array}$$ (9)

**Fig 1:**
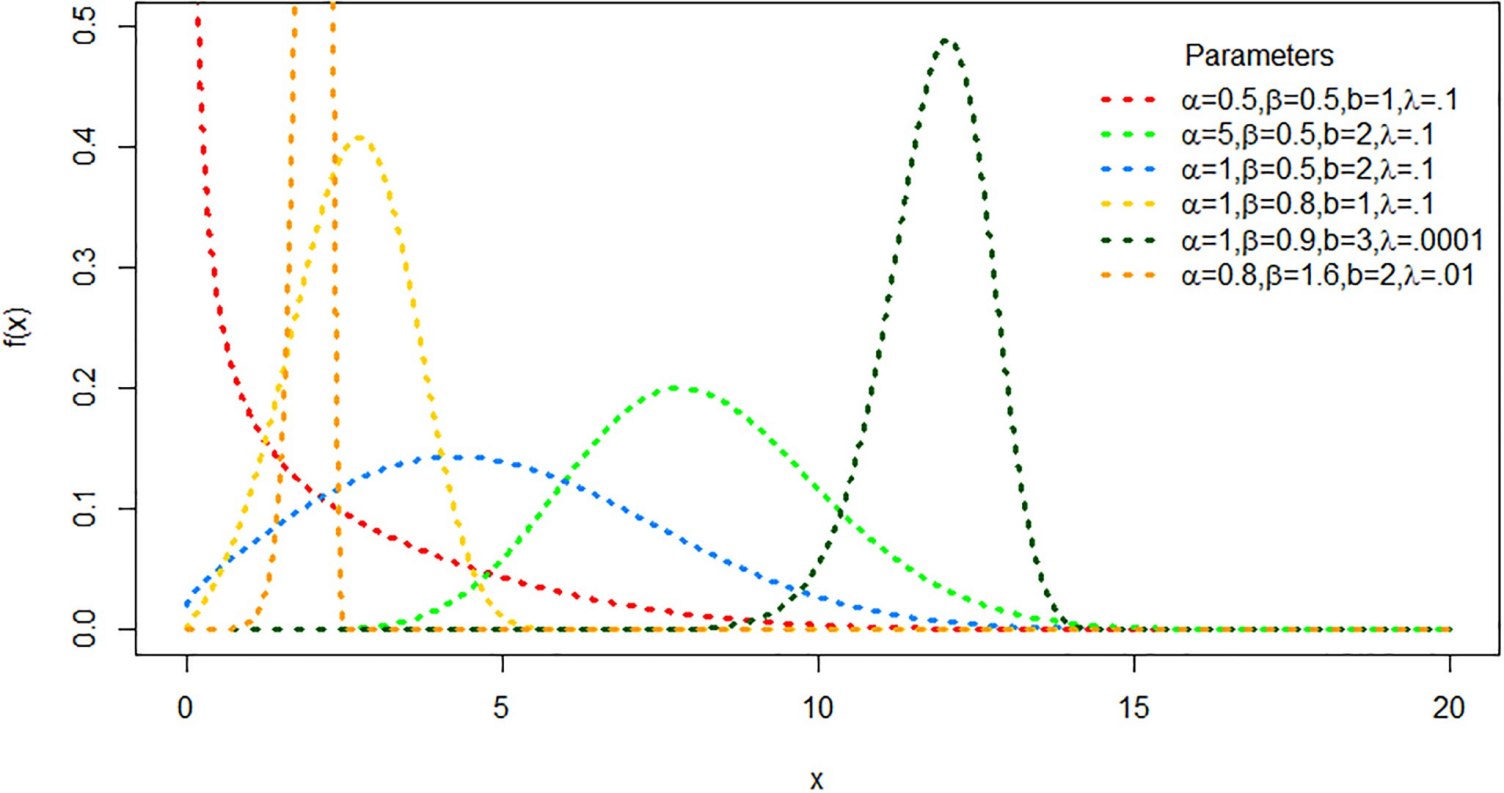
The PDF plots of TLEC distribution for various values of *α*, *β*, λ and *b*.

The PDF corresponding to Eq (9) is obtained as follows.

$$\begin{array}{c} \begin{array}{cc} f(x;\alpha ,\beta ,\lambda ,b) & =2\alpha b\beta \lambda x^{\beta -1}e^{x^{\beta }}e^{\lambda (1-e^{x^{\beta }})}{\left(1-e^{\lambda (1-e^{x^{\beta }})}\right)}^{b-1}\\ & \;\times \left[1-{\left(1-e^{\lambda (1-e^{x^{\beta }})}\right)}^{b}\right]{\left[1-{\left(1-{\left(1-e^{\lambda (1-e^{x^{\beta }})}\right)}^{b}\right)}^{2}\right]}^{\alpha -1}, \end{array} \end{array}$$ (10)

where λ is a scale parameter and *α*, *β* and b are shape parameters. Fig 1 shows various shapes of the CDF of the proposed distribution that can be almost symmetric, decreasing, right skewed and left skewed, which demonstrate its flexibility.

### 2.1 Alternative expression for the PDF of TLEC distribution

This section expands on the PDF of the TLEC distribution from Eq (8). Applying the following binomial series

(1-z)m=∑i=0∞(-1)i(mi)zi;m>0,|z|<1, (11)

we have

f(x;α)=2αg(x)∑i=0∞(α-1i)(-1)i[1-G(x)]2i+1. (12)

Substituting Eqs (5) and (6) in Eq (12), we have

f(x;α,β,λ,b)=2αbβλxβ-1exβeλ(1-exβ)(1-eλ(1-exβ))b-1×∑i=0∞(α-1i)(-1)i[1-[1-eλ(1-exβ)]b]2i+1. (13)

Using the binomial series in Eq (13), we get

f(x;α,β,λ,b)=2αbβλxβ-1exβeλ(1-exβ)∑i=0∞∑j=02i+1∑k=0∞(-1)i+j+k(α-1i)(2i+1j)×(b(j+1)-1k)eλ(k+1)(1-exβ). (14)

Applying the following expansion

$$\begin{array}{c} e^{x}=\sum_{l=0}^{\infty }\frac{x^{l}}{l!}, \end{array}$$ (15)

yields,

f(x;α,β,λ,b)=2αbβλ∑i,k,l=0∞∑j=02i+1∑m=0l(-1)i+j+k+m(α-1i)(2i+1j)(b(j+1)-1k)(lm)×(λ(k+1))ll!xβ-1e(m+1)xβ. (16)

Hence,

$$\begin{array}{c} \begin{array}{c} f\left(x;\alpha ,\beta ,\lambda ,b\right)=C_{1}x^{\beta -1}e^{\left(m+1\right)x^{\beta }}, \end{array} \end{array}$$ (17)

where C1=2αbβλ∑i,k,l=0∞∑j=02i+1∑m=0l(-1)i+j+k+m(α-1i)(2i+1j)(b(j+1)-1k)(lm)(λ(k+1))ll!.

When *b* = 1, the TLEC distribution becomes the Topp-Leone Chen distribution with CDF and PDF, respectively, as follows

$$\begin{array}{c} F\left(x;\alpha ,\beta ,\lambda \right)={\left[1-e^{\lambda (1-e^{x^{\beta }})}\right]}^{\alpha }{\left[1+e^{\lambda (1-e^{x^{\beta }})}\right]}^{\alpha },\;x>0,\alpha ,\lambda ,\beta >0, \end{array}$$ (18)

and

$$\begin{array}{c} f\left(x;\alpha ,\beta ,\lambda \right)=2\alpha \beta \lambda x^{\beta -1}e^{x^{\beta }}e^{2\lambda (1-e^{x^{\beta }})}{\left[1-e^{2\lambda (1-e^{x^{\beta }})}\right]}^{\alpha -1}. \end{array}$$ (19)

## 3 Sub-models of the TLEC distribution

The TLEC distribution is a highly flexible model that generalizes several well-known distributions depending on the values of its parameters. By setting specific parameters to particular values, the TLEC distribution simplifies into several important sub-models, each retaining a portion of the original model’s flexibility while serving different purposes in statistical modeling.

The following are the key sub-models derived from the TLEC distribution:

- **Exponentiated Chen Distribution:** When the Topp-Leone shape parameter *α* = 1, the TLEC distribution reduces to the Exponentiated Chen (EC) distribution, as introduced by [20]. In this case, the Topp-Leone generator is removed, and the resulting distribution offers flexibility through the additional shape parameter *b*, making it suitable for various applications in survival analysis and reliability studies.
- **Chen Distribution:** When both *α* = 1 and *b* = 1, the TLEC distribution further simplifies to the classical Chen distribution, initially developed by [14]. This distribution has been widely applied in reliability studies due to its ability to model data with bathtub-shaped and increasing hazard rates.
- **Topp-Leone Chen Distribution:** For the case when *b* = 1, the TLEC distribution simplifies to the Topp-Leone Chen (TLC) distribution. This sub-model eliminates the additional flexibility introduced by the exponentiation of the Chen distribution and leaves only the Topp-Leone generator applied to the Chen distribution. The TLC distribution maintains the flexibility in hazard rate shapes while offering simpler tail behavior, making it ideal for modeling data with J-shaped histograms.
- **Generalized Topp-Leone Exponentiated Chen Distribution:** With *α* > 1, λ > 0, *β* > 0, and *b* > 0, the full flexibility of the TLEC distribution is retained. This generalized form allows for diverse data modeling needs, providing flexibility in hazard rate shapes and tail behaviors.
- **Inverse Topp-Leone Exponentiated Chen Distribution:** By modifying the Chen distribution to include the inverse of the Exponentiated Chen component, the TLEC can be generalized to create models that fit different types of real-world data, particularly in reliability and survival studies. This sub-model is especially useful when the data exhibits decreasing failure rates or other non-standard hazard rate shapes.

These sub-models demonstrate the versatility of the TLEC distribution in adapting to various data characteristics. Depending on the complexity and behavior of the data, the model can be simplified to the Chen distribution or the Topp-Leone Chen distribution, or applied in its full generalized form for more complex data analysis needs.

### 3.1 Behavior of the TLEC distribution with different values of *b*

The TLEC distribution’s flexibility comes from the parameters *α*, *β*, and *b*. These affect the shape, skewness, and modality of the distribution. We explore key behaviors based on different values of these parameters.

#### 3.1.1 Unimodality and multimodality

When *α* and *β* are close, the TLEC distribution is unimodal. As the gap between them increases, particularly when *β* is large, the distribution may become multimodal, reflecting varying hazard rates.

#### 3.1.2 Special cases based on *b*

- **When** *b* = 1: The distribution simplifies to the Topp-Leone Chen distribution, reducing the complexity in the hazard rate.
- **When** *b* > 1: The distribution becomes more right-skewed with heavier tails, suited for modeling late events.
- **When** *b* < 1: The distribution is left-skewed with lighter tails, useful for modeling early failures.

#### 3.1.3 TLEC distribution for *b* = 1 and *b* > 1

For *b* = 1, the TLEC distribution simplifies to the Topp-Leone Chen (TLC) distribution, with the CDF and PDF given by:

$$\begin{array}{c} F\left(x;\alpha ,\lambda ,\beta \right)={\left[1-e^{\lambda (1-e^{x^{\beta }})}\right]}^{\alpha }{\left[2-e^{\lambda (1-e^{x^{\beta }})}\right]}^{\alpha }, \end{array}$$ (20)

$$\begin{array}{c} f\left(x;\alpha ,\lambda ,\beta \right)=2\alpha \beta \lambda x^{\beta -1}e^{x^{\beta }}e^{2\lambda (1-e^{x^{\beta }})}{\left[1-e^{2\lambda (1-e^{x^{\beta }})}\right]}^{\alpha -1}. \end{array}$$ (21)

For *b* > 1, the distribution becomes more flexible, with heavier tails. The CDF and PDF are given by:

$$\begin{array}{c} F\left(x;\alpha ,\lambda ,\beta ,b\right)={\left[1-{\left(1-e^{\lambda (1-e^{x^{\beta }})}\right)}^{b}\right]}^{\alpha }{\left[2-{\left(1-e^{\lambda (1-e^{x^{\beta }})}\right)}^{b}\right]}^{\alpha }, \end{array}$$ (22)

$$\begin{array}{c} f\left(x;\alpha ,\lambda ,\beta ,b\right)=2\alpha b\beta \lambda x^{\beta -1}e^{x^{\beta }}e^{\lambda (1-e^{x^{\beta }})}{\left(1-e^{\lambda (1-e^{x^{\beta }})}\right)}^{b-1}. \end{array}$$ (23)

## 4 Statistical properties

The moment generating function, r-*th* moment, hazard rate function, quantile, entropy and order statistics are some of the statistical properties of the new proposed model that are examined in this section.

### 4.1 Hazard rate function

The hazard rate function is defined as

$$\begin{array}{c} h\left(x\right)=\frac{f(x;\alpha ,\beta ,\lambda ,b)}{1-F(x;\alpha ,\beta ,\lambda ,b)}, \end{array}$$

where *F*(*x*) and *f*(*x*) are the CDF defined by Eq (9) and the PDF defined by Eq (10), respectively, hence, the hazard function for the TLEC distribution can be derived as follows:

$$\begin{array}{c} \begin{array}{cc} h(x)= & \frac{2\alpha b\beta \lambda x^{\beta -1}e^{x^{\beta }}e^{\lambda (1-e^{x^{\beta }})}{\left(1-e^{\lambda (1-e^{x^{\beta }})}\right)}^{b-1}\left[1-{\left(1-e^{\lambda (1-e^{x^{\beta }})}\right)}^{b}\right]}{1-{\left[1-{\left(1-{\left(1-e^{\lambda (1-e^{x^{\beta }})}\right)}^{b}\right)}^{2}\right]}^{\alpha }}\\ & \times {\left[1-{\left(1-{\left(1-e^{\lambda (1-e^{x^{\beta }})}\right)}^{b}\right)}^{2}\right]}^{\alpha -1}. \end{array} \end{array}$$ (24)

**Fig 2:**
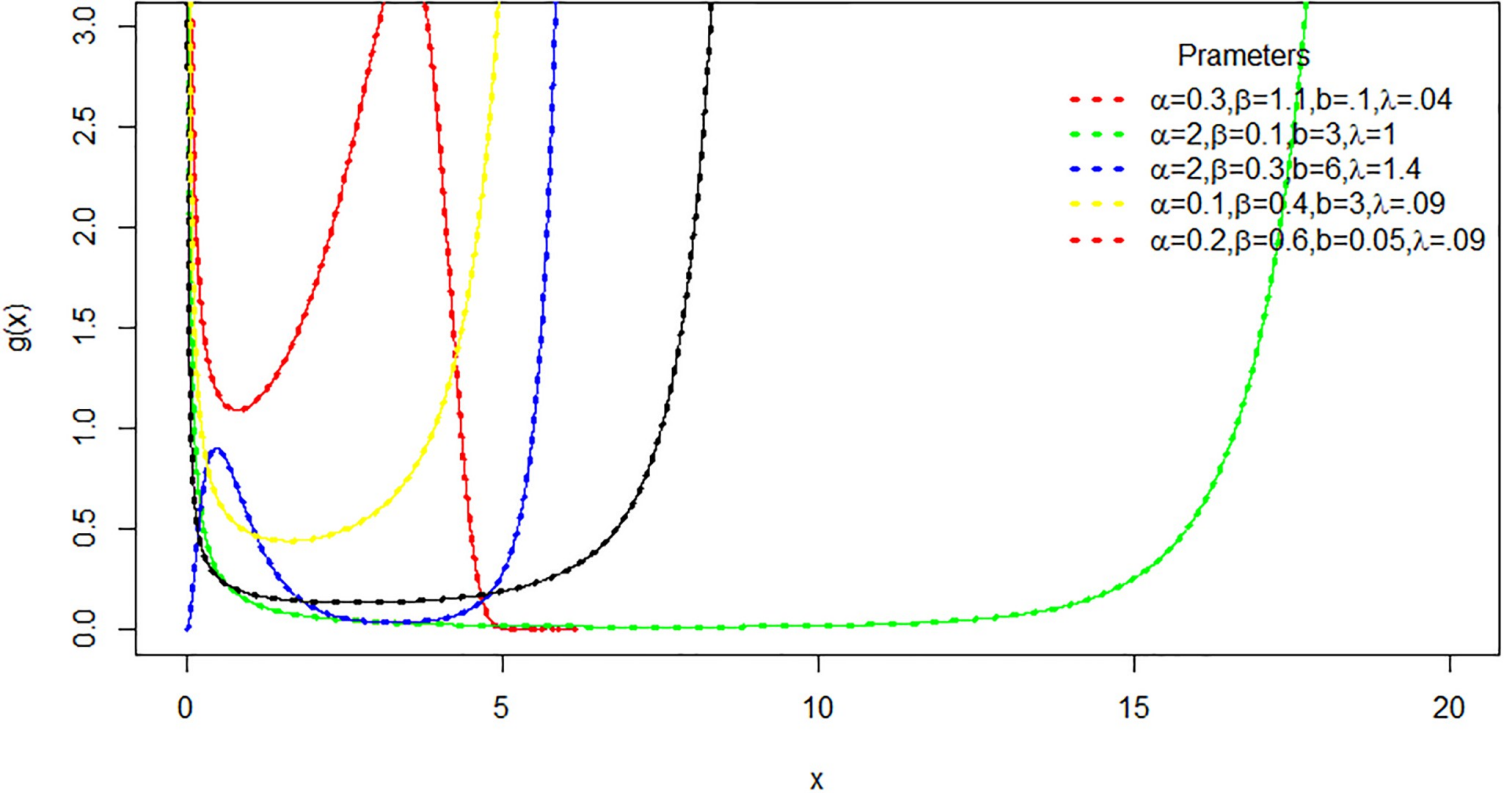
The plot of hazard rate function of TLEC distribution for various values of *α*, *β*, λ and *b*.

Fig 2 illustrates multiple shapes for the hazard rate function of the TLEC distribution. These shapes include bathtub, increasing and decreasing functions, which assure the flexibility of the distribution.

### 4.2 Moments

The *r*^*th*^ moment about the origin, *E*(*X*^*r*^), is defined as:

$$\mu_{r}=E\left(X^{r}\right)=\int_{0}^{\infty }x^{r}f\left(x\right)\;dx.$$

Substitute the PDF of the TLEC distribution:

$$f\left(x\right)=C_{1}x^{\beta -1}e^{\left(m+1\right)x^{\beta }},$$

into the moment formula:

$$\mu_{r}=\int_{0}^{\infty }x^{r}C_{1}x^{\beta -1}e^{\left(m+1\right)x^{\beta }}\;dx.$$

We now perform the variable substitution *u* = *x*^*β*^, so that *du* = *βx*^*β*−1^*dx*, and rewrite the integral as:

$$\mu_{r}=\frac{C_{1}}{\beta }\int_{0}^{\infty }u^{\frac{r}{\beta }}e^{-(1+m)u}\;du.$$

This integral is in the form of a gamma integral:

$$\int_{0}^{\infty }u^{a}e^{-bu}\;du=\frac{\Gamma (a+1)}{b^{a+1}},$$

which gives the moment:

$$\mu_{r}={\left(-1\right)}^{\frac{r}{\beta }+1}\frac{C_{1}}{\beta }\cdot \frac{\Gamma (\frac{r}{\beta }+1)}{{\left(1+m\right)}^{\frac{r}{\beta }+1}}.$$

### 4.3 Moment generating function

The moment generating function (MGF) for a random variable *X* is defined as:

$$M_{X}\left(t\right)=E\left(e^{tX}\right)=\int_{0}^{\infty }e^{tx}f\left(x\right)\;dx.$$

Substitute the PDF of the TLEC distribution:

$$f\left(x\right)=C_{1}x^{\beta -1}e^{\left(m+1\right)x^{\beta }},$$

we get:

$$M_{X}\left(t\right)=\int_{0}^{\infty }e^{tx}C_{1}x^{\beta -1}e^{\left(m+1\right)x^{\beta }}\;dx.$$

Performing the substitution *u* = *x*^*β*^, and using the series expansion for *e*^*tx*^, we have:

$$M_{X}\left(t\right)=\sum_{r=0}^{\infty }\frac{t^{r}}{r!}\int_{0}^{\infty }x^{r}f\left(x\right)\;dx.$$

Since $\int_{0}^{\infty }x^{r}f\left(x\right)\;dx$ is the *r*^*th*^ moment *μ*_*r*_, the MGF becomes:

$$M_{X}\left(t\right)=\sum_{r=0}^{\infty }\frac{t^{r}}{r!}\mu_{r}.$$

Using the expression for *μ*_*r*_:

$$\mu_{r}=\frac{C_{1}}{\beta }\cdot \frac{\Gamma (\frac{r}{\beta }+1)}{{\left(1+m\right)}^{\frac{r}{\beta }+1}},$$

the MGF is:

$$M_{X}\left(t\right)=\sum_{r=0}^{\infty }\frac{t^{r}}{r!}\cdot {\left(-1\right)}^{\frac{r}{\beta }+1}\frac{C_{1}}{\beta }\cdot \frac{\Gamma (\frac{r}{\beta }+1)}{{\left(1+m\right)}^{\frac{r}{\beta }+1}}.$$

### 4.4 Order statistics

The pdf of the k^*th*^ order statistic, *X*_*k*:*n*_ can be obtained using the following definition

$$\begin{array}{c} f_{k:n}\left(x\right)=\frac{n!}{(k-1)!(n-k)!}[f(x)]{\left[F(x)\right]}^{k-1}{\left[1-F(x)\right]}^{n-k}. \end{array}$$

Using the binomial series (11), yields

fk:n(x)=∑i=0∞(ni)(-1)in!(k-1)!(n-k)![f(x)][F(x)]i+k-1. (25)

Substituting the CDF in Eq (9) and PDF in Eq (10) of TLEC distribution in Eq (25) gives

fk:n(x)=∑i=0∞(ni)(-1)in!(k-1)!(n-k)![2αbβλxβ-1exβeλ(1-exβ)×(1-eλ(1-exβ))b-1[1-(1-eλ(1-exβ))b][[1-(1-((1-eλ(1-exβ))b))2]]α(i+k)-1, (26)

Applying Eq (11) into Eq (26), yields

fk:n(x)=∑i=0∞∑j=0∞(ni)(α(i+k)-1j)(-1)i+jn!(k-1)!(n-k)![2αbβλxβ-1exβeλ(1-exβ)·(1-eλ(1-exβ))b-1][1-((1-eλ(1-exβ))b)]2j+1. (27)

Therefore,

$$\begin{array}{c} \begin{array}{cc} f_{k:n}\left(x\right)= & C_{3}x^{\beta -1}e^{x^{\beta }}e^{\lambda (1-e^{x^{\beta }})}{\left[(1-e^{\lambda (1-e^{x^{\beta }})})\right]}^{b(m+1)-1}, \end{array} \end{array}$$ (28)

where C3=∑i=0∞∑j=0∞∑m=0∞(ni)(α(i+k)-1j)(2j+1m)(-1)i+j+mn!(k-1)!(n-k)![2αbβλ].

#### 4.4.1 Comparison between TLEC order statistic and Beta distribution

In this section, we investigate whether the TLEC distribution exhibits characteristics of the Beta distribution by utilizing a transformation technique. The goal is to assess whether the order statistic of the TLEC distribution can be related to the Beta distribution.

We begin with the given PDF of the *k*^*th*^ order statistics of the TLEC distribution in Eq (28)

$$f\left(x\right)=C_{3}x^{\beta -1}e^{x^{\beta }}e^{\lambda (1-e^{x^{\beta }})}{\left[1-e^{\lambda (1-e^{x^{\beta }})}\right]}^{b(m+1)-1}.$$

Using the transformation technique, let:

$$y=e^{\lambda }\left(1-e^{x^{\beta }}\right),$$

therefore,

$$x={\left[\text{log}\;(1-\frac{1}{\lambda }\;\text{log}\;y)\right]}^{\frac{1}{\beta }}\cdot$$

Next, we calculate the derivative of *x* with respect to *y*:

$$|\frac{dx}{dy}|=\frac{1}{\beta }{\left[\text{log}\;(1-\frac{1}{\lambda }\;\text{log}\;y)\right]}^{\frac{1}{\beta }-1}\frac{1}{1-\frac{1}{\lambda }\;\text{log}\;y}|\frac{-1}{\lambda y}|.$$

Thus, the PDF of the random variable *y* is:

$$f\left(y\right)=f\left(x\right)|\frac{dx}{dy}|\cdot$$

Substituting into the expression for *f*(*y*):

$$\begin{array}{c} f\left(y\right)=C_{3}{\left[\text{log}\;{\left(1-\frac{1}{\lambda }\;\text{log}\;y\right)}^{\frac{1}{\beta }}\right]}^{\beta -1}e^{\text{log}(1-\frac{1}{\lambda }\;\text{log}\;y)}e^{\text{log}\;y}{\left[1-y\right]}^{b(m+1)-1}\\ \times \frac{1}{\beta }{\left[\text{log}\;(1-\frac{1}{\lambda }\;\text{log}\;y)\right]}^{\frac{1}{\beta }-1}\frac{1}{\lambda y(1-\frac{1}{\lambda }log\left(y\right))}\cdot \end{array}$$

Recognizing this as a Beta distribution:

$$f\left(y\right)=\frac{C_{3}}{\beta \lambda }\beta \left(1,b\left(m+1\right)\right)\cdot$$

Through this transformation, we see that the TLEC distribution can exhibit Beta-like behavior. This relationship highlights that, under appropriate parameterization, the order statistics of the TLEC distribution can be mapped to a Beta distribution, demonstrating a mathematical connection between the two distributions.

### 4.5 Renyi entropy

The Renyi Entropy is defined as follows:

$$\begin{array}{c} \begin{array}{c} RE_{X}\left(\theta \right)=\frac{1}{1-\theta }\text{log}(\int_{0}^{\infty }f{\left(x\right)}^{\theta })dx,\;\theta >0,\theta \neq 1, \end{array} \end{array}$$

and hence the Renyi Entropy is obtained from the PDF in Eq (10) as

$$\begin{array}{c} RE_{X}\left(\theta \right)=C_{4}\frac{\Gamma (\frac{\theta (\beta -1)+1}{\beta })}{{\left(\theta +l\right)}^{\frac{\theta (\beta -1)+1}{\beta }}}, \end{array}$$ (29)

where

C4=(2αbβλ)θ∑i=0∞∑j=0∞∑k=0∞∑l=0∞(θ(α-1)i)(2i+θj)(bj+θ(b-1)k)(-1)i+j+k+l-θ(β-1)+1βl!(λ(k+θ))leλ(k+θ).

### Proof

We begin with the definition of Renyi Entropy for a random variable *X* with probability density function (PDF) *f*(*x*):

$$\begin{array}{c} RE_{X}\left(\theta \right)=\frac{1}{1-\theta }\text{log}(\int_{0}^{\infty }f{\left(x\right)}^{\theta }\;dx),\;\theta >0,\;\theta \neq 1, \end{array}$$ (30)

where *f*(*x*) is the PDF of the Topp-Leone Exponentiated Chen (TLEC) distribution given by:

$$\begin{array}{cc} f(x) & =2\alpha b\beta \lambda x^{\beta -1}e^{x^{\beta }}e^{\lambda (1-e^{x^{\beta }})}{\left(1-e^{\lambda (1-e^{x^{\beta }})}\right)}^{b-1}\\ & \;\times [1-{\left(1-e^{\lambda (1-e^{x^{\beta }})}\right)}^{b}]{\left[1-{\left(1-{\left(1-e^{\lambda (1-e^{x^{\beta }})}\right)}^{b}\right)}^{2}\right]}^{\alpha -1}. \end{array}$$ (31)

Step 1: Expression for *f*(*x*)^*θ*^

We first compute *f*(*x*)^*θ*^, which is required for the integral in the Renyi Entropy formula:

$$\begin{array}{c} f{\left(x\right)}^{\theta }={\left(2\alpha b\beta \lambda x^{\beta -1}e^{x^{\beta }}e^{\lambda (1-e^{x^{\beta }})}\right)}^{\theta }{\left(1-e^{\lambda (1-e^{x^{\beta }})}\right)}^{\theta (b-1)}{\left[1-{\left(1-e^{\lambda (1-e^{x^{\beta }})}\right)}^{b}\right]}^{\theta }. \end{array}$$ (32)

This gives:

$$\begin{array}{c} f{\left(x\right)}^{\theta }={\left(2\alpha b\beta \lambda \right)}^{\theta }x^{\theta (\beta -1)}e^{\theta x^{\beta }}e^{\theta \lambda (1-e^{x^{\beta }})}{\left(1-e^{\lambda (1-e^{x^{\beta }})}\right)}^{\theta (b-1)}{\left[1-{\left(1-e^{\lambda (1-e^{x^{\beta }})}\right)}^{b}\right]}^{\theta }. \end{array}$$ (33)

Step 2: Integration of *f*(*x*)^*θ*^

Now, we compute the integral of *f*(*x*)^*θ*^:

$$\begin{array}{cc} \int_{0}^{\infty }f{\left(x\right)}^{\theta }\;dx & ={\left(2\alpha b\beta \lambda \right)}^{\theta }\int_{0}^{\infty }x^{\theta (\beta -1)}e^{\theta x^{\beta }}e^{\theta \lambda (1-e^{x^{\beta }})}\\ & \;\times {\left(1-e^{\lambda (1-e^{x^{\beta }})}\right)}^{\theta (b-1)}{\left[1-{\left(1-e^{\lambda (1-e^{x^{\beta }})}\right)}^{b}\right]}^{\theta }\;dx. \end{array}$$ (34)

This integral can be solved using series expansion techniques for the exponential and power terms. By expanding the terms and grouping powers of *x*, we obtain the following form:

$$\begin{array}{c} \int_{0}^{\infty }f{\left(x\right)}^{\theta }\;dx=C_{4}\cdot \frac{\Gamma (\frac{\theta (\beta -1)+1}{\beta })}{{\left(\theta +l\right)}^{\frac{\theta (\beta -1)+1}{\beta }}}, \end{array}$$ (35)

where *C*_4_ is a constant involving the summation terms arising from the series expansion, given by:

C4=(2αbβλ)θ∑i=0∞∑j=0∞∑k=0∞∑l=0∞(θ(α-1)i)(2i+θj)(bj+θ(b-1)k)×(-1)i+j+k+l-θ(β-1)+1βl!(λ(k+θ))leλ(k+θ). (36)

Step 3: Final Expression for Renyi Entropy

Substituting the result of the integral into the definition of Renyi Entropy in Eq (30), we get:

$$\begin{array}{c} RE_{X}\left(\theta \right)=\frac{1}{1-\theta }\text{log}(C_{4}\cdot \frac{\Gamma (\frac{\theta (\beta -1)+1}{\beta })}{{\left(\theta +l\right)}^{\frac{\theta (\beta -1)+1}{\beta }}}). \end{array}$$ (37)

Simplifying the logarithmic expression:

$$\begin{array}{c} RE_{X}\left(\theta \right)=C_{4}\cdot \frac{\Gamma (\frac{\theta (\beta -1)+1}{\beta })}{{\left(\theta +l\right)}^{\frac{\theta (\beta -1)+1}{\beta }}}. \end{array}$$ (38)

Thus, the result in Equation (RE) is proven.

### 4.6 Quantile

The quantile function of the TLEC distribution can be derived using Eq (7) as shown below:

$$\begin{array}{c} P^{\frac{1}{\alpha }}=1-{\left(\bar{G}\left(x\right)\right)}^{2},\;x>0,\alpha >0, \end{array}$$ (39)

where, *P* ∈ *U*(0, 1). Substituting Eq (5) in Eq (39), and after some algebraic operations, the quantile function of the random variable *X* can be obtained in the following form

$$\begin{array}{c} Q_{P}={\left[\text{log}[1-\frac{1}{\lambda }\text{log}[1-{\left(1-{\left(1-P^{\frac{1}{\alpha }}\right)}^{\frac{1}{2}}\right)}^{\frac{1}{b}}]]\right]}^{\frac{1}{\beta }}. \end{array}$$ (40)

#### 4.6.1 Lower quartile, median and upper quartile

The quantile function of the TLEC distribution is given by Eq (40):

$$Q_{P}={\left[\text{log}\left[1-\frac{1}{\lambda }\text{log}\left[1-{\left(1-{\left(1-P^{\frac{1}{\alpha }}\right)}^{\frac{1}{2}}\right)}^{\frac{1}{b}}\right]\right]\right]}^{\frac{1}{\beta }},$$

where *P* ∈ (0, 1) represents the percentile.

To obtain the lower quartile (*Q*_1_), median (*Q*_2_), and upper quartile (*Q*_3_), we substitute the respective values of *P* = 0.25, *P* = 0.50, and *P* = 0.75.

- **Lower Quartile** (*Q*_1_) **for (P = 0.25)**
$$Q_{0.25}={\left[\text{log}\left[1-\frac{1}{\lambda }\text{log}\left[1-{\left(1-{\left(1-0.25^{\frac{1}{\alpha }}\right)}^{\frac{1}{2}}\right)}^{\frac{1}{b}}\right]\right]\right]}^{\frac{1}{\beta }}.$$
- **Median** (*Q*_2_) **for** *P* = 0.50
$$Q_{0.50}={\left[\text{log}\left[1-\frac{1}{\lambda }\text{log}\left[1-{\left(1-{\left(1-0.50^{\frac{1}{\alpha }}\right)}^{\frac{1}{2}}\right)}^{\frac{1}{b}}\right]\right]\right]}^{\frac{1}{\beta }}.$$
- **Upper Quartile** (*Q*_3_) **for** *P* = 0.75
$$Q_{0.75}={\left[\text{log}\left[1-\frac{1}{\lambda }\text{log}\left[1-{\left(1-{\left(1-0.75^{\frac{1}{\alpha }}\right)}^{\frac{1}{2}}\right)}^{\frac{1}{b}}\right]\right]\right]}^{\frac{1}{\beta }}.$$

By substituting the values of the parameters *α*, *β*, λ, and *b*, the lower quartile, median, and upper quartile of the TLEC distribution can be calculated.

## 5 Maximum likelihood estimation

Let *x*_1_, *x*_2_, …*x*_*n*_ be a random sample of size *n* from the TLEC distribution with the PDF given by Eq (10). The likelihood function is given by

$$\begin{array}{c} \begin{array}{cc} L(x;\alpha ,\lambda ,\beta ,b) & =\prod_{i=1}^{n}2\alpha b\beta \lambda x^{\beta -1}e^{x^{\beta }}e^{\lambda (1-e^{x^{\beta }})}{\left(1-e^{\lambda (1-e^{x^{\beta }})}\right)}^{b-1}\left[1-{\left(1-e^{\lambda (1-e^{x^{\beta }})}\right)}^{b}\right]\\ & \cdot {\left[1-{\left(1-{\left(1-e^{\lambda (1-e^{x^{\beta }})}\right)}^{b}\right)}^{2}\right]}^{\alpha -1}. \end{array} \end{array}$$

Therefore, the log likelihood function is

$$\begin{array}{c} \begin{array}{cc} logL(x;\alpha ,\lambda ,\beta ,b) & =nlog\left(2\alpha b\beta \lambda \right)+\left(\beta -1\right)\sum_{i=1}^{n}logx_{i}+\sum_{i=1}^{n}x_{i}^{\beta }+\lambda \sum_{i=1}^{n}\left(1-e^{x_{i}^{\beta }}\right)\\ & +\left(b-1\right)\sum_{i=1}^{n}log\left(1-e^{\lambda (1-e^{x_{i}^{\beta }})}\right)+\sum_{i=1}^{n}log\left[1-{\left(1-e^{\lambda (1-e^{x_{i}^{\beta }})}\right)}^{b}\right]\\ & +\left(\alpha -1\right)\sum_{i=1}^{n}log\left[1-{\left(1-{\left(1-e^{\lambda (1-e^{x_{i}^{\beta }})}\right)}^{b}\right)}^{2}\right]. \end{array} \end{array}$$ (41)

The first derivatives of Eq (41) with respect to *α*, *β*, λ and *b*, respectively, are obtained as follows:

$$\begin{array}{c} \begin{array}{cc} \frac{\partial \ell }{\partial \alpha }= & \frac{n}{\alpha }+\sum_{i=1}^{n}\text{log}\left(1-{\left(1-{\left(1-\text{exp}\left(\lambda \left(1-e^{x_{i}^{\beta }}\right)\right)\right)}^{b}\right)}^{2}\right), \end{array} \end{array}$$ (42)

$$\begin{array}{c} \begin{array}{cc} \frac{\partial \ell }{\partial \beta }= & \frac{n}{\beta }+\sum_{i=1}^{n}\text{log}\left(x_{i}\right)+\sum_{i=1}^{n}x_{i}^{\beta }\text{log}\left(x_{i}\right)-\lambda \sum_{i=1}^{n}x_{i}e^{x_{i}^{\beta }}\\ & +\lambda \sum_{i=1}^{n}\frac{\left(b-1\right)x_{i}\text{exp}\left(\lambda \left(1-e^{x_{i}^{\beta }}\right)\right)e^{x_{i}^{\beta }}}{1-\text{exp}\left(\lambda \left(1-e^{x_{i}^{\beta }}\right)\right)}\\ & -\lambda b\sum_{i=1}^{n}\frac{x_{i}\text{exp}\left(\lambda {\left(1-e^{x_{i}^{\beta }}\right)}^{b}\right){\left(1-e^{x_{i}^{\beta }}\right)}^{b-1}e^{x_{i}^{\beta }}}{1-\left(1-\text{exp}\left(\lambda {\left(1-e^{x_{i}^{\beta }}\right)}^{b}\right)\right)}\\ & +\sum_{i=1}^{n}\frac{2\lambda b\left(\alpha -1\right){\left(1-\text{exp}\left(\lambda \left(1-e^{x_{i}^{\beta }}\right)\right)\right)}^{b-1}\text{exp}\left(\lambda \left(1-e^{x_{i}^{\beta }}\right)\right)e^{x_{i}^{\beta }}x_{i}}{1-{\left(1-{\left(1-\text{exp}\left(\lambda \left(1-e^{x_{i}^{\beta }}\right)\right)\right)}^{b}\right)}^{2}}\\ & \times \left(1-{\left(1-\text{exp}\left(\lambda \left(1-e^{x_{i}^{\beta }}\right)\right)\right)}^{b}\right). \end{array} \end{array}$$ (43)

$$\begin{array}{c} \begin{array}{cc} \frac{\partial \ell }{\partial \lambda }= & \frac{n}{\lambda }+\sum_{i=1}^{n}(1-e^{x_{i}^{\beta }})\\ & -\sum_{i=1}^{n}\frac{\left(b-1)\text{exp}(\lambda (1-e^{x_{i}^{\beta }}))(1-e^{x_{i}^{\beta }}\right)}{1-\text{exp}(\lambda (1-e^{x_{i}^{\beta }}))}\\ & +\sum_{i=1}^{n}\frac{\text{exp}(\lambda {\left(1-e^{x_{i}^{\beta }}\right)}^{b}){\left(1-e^{x_{i}^{\beta }}\right)}^{b}}{1-(1-\text{exp}(\lambda {\left(1-e^{x_{i}^{\beta }}\right)}^{b}))}\\ & -\sum_{i=1}^{n}\frac{2(\alpha -1)b{\left(1-\text{exp}(\lambda (1-e^{x_{i}^{\beta }}))\right)}^{b-1}\text{exp}(\lambda (1-e^{x_{i}^{\beta }}))}{1-{\left(1-{\left(1-\text{exp}(\lambda (1-e^{x_{i}^{\beta }}))\right)}^{b}\right)}^{2}}\\ & \times (1-e^{x_{i}^{\beta }}). \end{array} \end{array}$$ (44)

$$\begin{array}{c} \begin{array}{cc} \frac{\partial \ell }{\partial b}= & \frac{n}{b}+\sum_{i=1}^{n}\text{log}\left(1-\text{exp}\left(\lambda \left(1-e^{x_{i}^{\beta }}\right)\right)\right)\\ & +\sum_{i=1}^{n}\frac{\text{exp}\left(\lambda {\left(1-e^{x_{i}^{\beta }}\right)}^{b}\right)\lambda \left(1-{\left(e^{x_{i}^{\beta }}\right)}^{b}\right)\text{log}\left(1-e^{x_{i}^{\beta }}\right)}{1-\left(1-\text{exp}\left(\lambda {\left(1-e^{x_{i}^{\beta }}\right)}^{b}\right)\right)}\\ & +\sum_{i=1}^{n}\frac{2\left(\alpha -1\right){\left(1-\text{exp}\left(\lambda \left(1-e^{x_{i}^{\beta }}\right)\right)\right)}^{b}\text{log}(1-\text{exp}\left(\lambda \left(1-e^{x_{i}^{\beta }}\right)\right)}{1-{\left(1-{\left(1-\text{exp}\left(\lambda \left(1-e^{x_{i}^{\beta }}\right)\right)\right)}^{b}\right)}^{2}}\\ & \;\times \left(1-{\left(1-\text{exp}\left(\lambda \left(1-e^{x_{i}^{\beta }}\right)\right)\right)}^{b}\right). \end{array} \end{array}$$ (45)

Setting the previous equations to zero and calculating them all numerically yields the maximum likelihood estimates (MLEs) of the four TLEC distribution parameters. The 4x4 observed information matrix, $I(\hat{\eta })$, is required to construct 100(1-*γ*)% approximate confidence intervals for *η* = (*α*, *β*, λ, *b*)^*T*^ based on the multivariate normal distribution *N*_4_(0, *I*^−1^), where *γ* is the confidence coefficient. Its components have been written in details in Appendix 8.

## 6 Simulation studies

In this study, 5000 random samples of sizes *n* = 50, 100, 200 and 300 are generated from the TLEC distribution using the quantile function given by Eq (40) to obtain the MLE of the parameters *α*, λ, *β* and *b*. The Nelder-Mead optimization method in R software is used. Three cases are considered for the true parameters as follows:

**Case1:** *α* = 0.6, λ = 0.9, *β* = 0.5, *b* = 0.3.
**Case2:** *α* = 0.7, λ = 0.4, *β* = 0.1, *b* = 0.3.
**Case3:** *α* = 1.2, λ = 1.5, *β* = 1.3, *b* = 1.4.

Let $\hat{\eta }$ represents the MLE for each parameter in TLEC distribution. To evaluate the accuracy of the MLE of the parameter *η*, average Bias and Mean squared error (MSE) are calculated, which are defined as follows:

$$\begin{array}{c} \text{MSE}\left(\hat{\eta }\right)=\frac{\sum_{i=1}^{5000}{\left(\hat{\eta }-\eta \right)}^{2}}{5000}. \end{array}$$ (46)

and

$$\begin{array}{c} \text{Average}\;\text{Bias}\left(\hat{\eta }\right)=\frac{\sum_{i=1}^{5000}\hat{\eta_{i}}}{5000}-\eta , \end{array}$$ (47)

**Eq (47)** defines the average bias, which measures the difference between the estimated parameter values and the true parameter. This formula provides insight into the accuracy of the MLEs, while **Eq (46)** defines the MSE, which evaluates the precision of the estimates.

Additionally, the approximate two-sided confidence intervals are calculated using 95% confidence level. The values for the MLE of the TLEC distribution parameters are shown in Tables 1 and 2, accompanied by the related average bias, MSE and confidence intervals. It is obvious that the MLEs get closer to the initial values of the parameters, as well as the average Bias, MSE and the length of the interval decrease as the sample size increases in almost all cases. Tables 3–5 provide the coverage probabilities for the same sample sizes and parameters presented in Tables 1, 2 and 6. It is obvious that, when the sample size increase the probability of the coverage increase which demonstrates the overall effectiveness of the interval estimation method.

**Table 1:** Parameters estimation for sample sizes *n* = 50, 100, 200, 300 when the true values of the parameters *α* = 0.6, λ = 0.9, *β* = 0.5, *b* = 0.3.

*n*	Parameters	Estimate	Average Bias	MSE	lower limit	upper limit	Length
50	*α*	0.7658	0.1658	0.0785	0.3986	1.1330	0.7344
	λ	1.0154	0.1154	0.0275	0.7123	1.3185	0.6062
	*β*	0.5395	0.0395	0.0081	0.4167	0.6624	0.2457
	*b*	0.2531	-0.0469	0.0139	0.1184	0.3878	0.2694
100	*α*	0.7473	0.1473	0.0606	0.4227	1.0719	0.6492
	λ	0.9889	0.0889	0.0172	0.8301	1.1477	0.3176
	*β*	0.5329	0.0329	0.0037	0.4490	0.6168	0.1678
	*b*	0.2637	-0.0363	0.0070	0.1400	0.3873	0.2473
200	*α*	0.5532	-0.0468	0.0107	0.4011	0.7054	0.3043
	λ	0.9593	0.0593	0.0202	0.7470	1.1715	0.4244
	*β*	0.5100	0.0100	0.0135	0.3196	0.7004	0.3807
	*b*	0.3469	0.0469	0.0133	0.1734	0.5204	0.3470
300	*α*	0.6396	0.0396	0.0059	0.5314	0.7478	0.2163
	λ	0.9195	0.0195	0.0026	0.8429	0.9962	0.1533
	*β*	0.4713	-0.0287	0.0049	0.3660	0.5766	0.2107
	*b*	0.3180	0.0180	0.0042	0.2155	0.4204	0.2049

**Table 2:** Parameters estimation for sample sizes *n* = 50, 100, 200, 300 when the true values of the parameters *α* = 0.7, λ = 0.4, *β* = 0.1, *b* = 0.3.

*n*	Parameters	Estimate	Average Bias	MSE	lower limit	upper limit	Length
50	*α*	0.7613	0.0613	0.1445	0.3654	1.1571	0.7917
	λ	0.3324	-0.0676	0.0264	0.1993	0.4655	0.2662
	*β*	0.1147	0.0147	0.0031	0.0596	0.1697	0.1101
	*b*	0.2383	-0.0617	0.0126	0.1264	0.3502	0.2238
100	*α*	0.6936	0.0036	0.1321	0.0957	1.2915	1.1958
	λ	0.3157	-0.0443	0.0149	0.1288	0.5027	0.3739
	*β*	0.1089	0.0339	0.0018	0.0676	0.1501	0.0825
	*b*	0.2484	-0.0516	0.0097	0.1099	0.3868	0.2769
200	*α*	0.6642	-0.0258	0.0729	0.2219	1.1064	0.8845
	λ	0.3290	-0.0310	0.0043	0.2337	0.4242	0.1905
	*β*	0.1010	0.0260	0.0007	0.0938	0.1081	0.0143
	*b*	0.2825	-0.0175	0.0028	0.2004	0.3646	0.1642
300	*α*	0.7255	0.0355	0.0230	0.4828	0.9681	0.4852
	λ	0.3260	-0.0340	0.0069	0.2012	0.4508	0.2496
	*β*	0.1018	0.0268	0.0008	0.0914	0.1123	0.0208
	*b*	0.2740	-0.0260	0.0028	0.1986	0.3495	0.1510

**Table 3:** Coverage probability for sample sizes *n* = 50, 100, 200, 300 when the true values of the parameters *α* = 0.6, λ = 0.9, *β* = 0.5, *b* = 0.3.

*n*	*α*	λ	*β*	*b*
50	0.90	0.88	0.87	0.85
100	0.93	0.92	0.91	0.90
200	0.95	0.94	0.94	0.93
300	0.96	0.95	0.95	0.94

**Table 4:** Coverage probability for sample sizes *n* = 50, 100, 200, 300 when the true values of the parameters *α* = 0.7, λ = 0.4, *β* = 0.1, *b* = 0.3.

*n*	*α*	λ	*β*	*b*
50	0.88	0.86	0.84	0.81
100	0.91	0.90	0.89	0.87
200	0.94	0.93	0.92	0.91
300	0.96	0.95	0.95	0.94

**Table 5:** Coverage probability for sample sizes *n* = 50, 100, 200, 300 when the true values of the parameters *α* = 1.2, λ = 1.5, *β* = 1.3, *b* = 1.4.

*n*	*α*	λ	*β*	*b*
50	0.83	0.80	0.79	0.78
100	0.88	0.85	0.84	0.82
200	0.91	0.90	0.89	0.88
300	0.94	0.93	0.93	0.92

**Table 6:** Parameters estimation for sample sizes *n* = 50, 100, 200, 300 when the true values of the parameters *α* = 1.2, λ = 1.5, *β* = 1.3, *b* = 1.4.

*n*	Parameters	Estimate	Average Bias	MSE	Lower Limit	Upper Limit	Length
50	*α*	1.4325	0.2325	0.0521	0.8523	2.0127	1.1604
	λ	1.6852	0.1852	0.0365	1.0914	2.2790	1.1876
	*β*	1.3789	0.0789	0.0124	1.1226	1.6352	0.5126
	*b*	1.2975	-0.1025	0.0231	0.8315	1.7635	0.9320
100	*α*	1.2803	0.0803	0.0302	0.9732	1.5874	0.6142
	λ	1.5721	0.0721	0.0218	1.3174	1.8268	0.5094
	*β*	1.3335	0.0335	0.0095	1.2116	1.4554	0.2438
	*b*	1.3847	-0.0153	0.0106	1.2172	1.5522	0.3350
200	*α*	1.2356	0.0356	0.0152	1.0987	1.3725	0.2738
	λ	1.5389	0.0389	0.0087	1.4395	1.6383	0.1988
	*β*	1.3107	0.0107	0.0054	1.2441	1.3773	0.1332
	*b*	1.4075	0.0075	0.0051	1.3126	1.5025	0.1899
300	*α*	1.2152	0.0152	0.0049	1.1547	1.2757	0.1210
	λ	1.5235	0.0235	0.0032	1.4578	1.5892	0.1314
	*β*	1.2989	-0.0011	0.0026	1.2451	1.3528	0.1077
	*b*	1.4126	0.0126	0.0021	1.3625	1.4627	0.1002

The details of the tables for all the three cases are provided with examples as follows:

**Case 1**: *α* = 0.6, λ = 0.9, *β* = 0.5, *b* = 0.3

In Table 1, as the sample size increases from 50 to 300, the accuracy of the MLEs for the parameters *α*, λ, *β*, and *b* improves significantly. For instance, the estimate for *α* is much closer to the true value (0.6) at *n* = 300 (0.6396) compared to *n* = 50 (0.7658).

- **Bias:** The average bias decreases as the sample size grows, indicating more accurate estimates. For example, the bias for *α* drops from 0.1658 at *n* = 50 to 0.0204 at *n* = 300.
- **MSE:** The mean squared error (MSE) also decreases with larger sample sizes, reflecting improved precision. For instance, the MSE for λ decreases from 0.0275 (*n* = 50) to 0.0026 (*n* = 300).
- **Confidence Interval Length:** The length of the confidence intervals becomes shorter as sample sizes increase, indicating more reliable estimates. For example, the interval length for *β* decreases from 0.2457 (*n* = 50) to 0.2107 (*n* = 300).

**Case 2**: *α* = 0.7, λ = 0.4, *β* = 0.1, *b* = 0.3

In Table 2, similar trends are observed as the sample size increases. The estimates for the parameters improve, and both bias and MSE decrease. For example, the estimate for *b* gets closer to the true value (0.3) as the sample size grows, with the bias decreasing from -0.0617 at *n* = 50 to -0.0260 at *n* = 300.

- **Bias and MSE:** As the sample size increases, the bias and MSE decrease for all parameters, showing improved accuracy and precision of the estimates.
- **Confidence Interval Length:** The confidence intervals become narrower with larger sample sizes, indicating higher confidence in the parameter estimates.

**Case 3**: *α* = 1.2, λ = 1.5, *β* = 1.3, *b* = 1.4

In Table 6, when all parameters are greater than one, the accuracy of the estimates improves with increasing sample size. For example, the estimate for λ at *n* = 300 is 1.5235, close to the true value of 1.5. Similarly, the confidence interval length for *b* reduces from 0.9320 (*n* = 50) to 0.1002 (*n* = 300), indicating more reliable estimates as sample sizes grow.

### 6.1 Coverage probability

In all three cases in Tables (3–5), coverage probability increases with the sample size. In Case 1, *α*’s coverage probability reaches 0.96 at *n* = 300, improving from 0.90 at *n* = 50. Case 2 and Case 3 show similar improvements, with coverage probability nearing 0.96 for larger *n*. Larger samples result in better interval estimation and higher confidence in the estimates.

### 6.2 Simulation results for parameter estimation when *b* = 1 and *b* = 2

We present the simulation results for parameter estimation of the TLEC distribution with *b* = 1 and *b* = 2. The study involved 5000 random samples of sizes *n* = 50, 100, 200, 300 to estimate *α*, λ, *β*, and *b*.

#### 6.2.1 Results for *b* = 1 (Table 7)

**Table 7:** Parameters estimation for sample sizes *n* = 50, 100, 200, 300 when the true values of the parameters *α* = 0.6, λ = 0.9, *β* = 0.5, *b* = 1.

*n*	Parameters	Estimate	Average Bias	MSE	Lower Limit	Upper Limit	Length
50	*α*	0.7113	0.1113	0.0456	0.5121	0.9105	0.3984
	λ	0.9349	0.0349	0.0102	0.7832	1.0866	0.3034
	*β*	0.5057	0.0057	0.0026	0.4635	0.5478	0.0843
	*b*	1.0005	0.0005	0.0000	0.9023	1.0987	0.1964
100	*α*	0.7016	0.1016	0.0425	0.5809	0.8223	0.2414
	λ	0.9223	0.0223	0.0079	0.8319	1.0127	0.1808
	*β*	0.5039	0.0039	0.0013	0.4712	0.5366	0.0654
	*b*	1.0003	0.0003	0.0000	0.9517	1.0489	0.0971
200	*α*	0.6765	0.0765	0.0215	0.6123	0.7407	0.1284
	λ	0.9104	0.0104	0.0039	0.8721	0.9487	0.0766
	*β*	0.5024	0.0024	0.0011	0.4898	0.5150	0.0252
	*b*	1.0001	0.0001	0.0000	0.9721	1.0281	0.0560
300	*α*	0.6547	0.0547	0.0089	0.6201	0.6893	0.0691
	λ	0.9058	0.0058	0.0026	0.8863	0.9253	0.0389
	*β*	0.5009	0.0009	0.0005	0.4945	0.5074	0.0129
	*b*	1.0000	0.0000	0.0000	0.9825	1.0175	0.0350

When *b* = 1, the distribution simplifies to the Topp-Leone Chen distribution. As *n* increases, the parameter estimates improve, with average bias and MSE decreasing. For example, the bias for *α* reduces from 0.1113 at *n* = 50 to 0.0547 at *n* = 300. Confidence intervals also become narrower, indicating increased precision.

#### 6.2.2 Results for *b* = 2 (Table 8)

**Table 8:** Parameters estimation for sample sizes *n* = 50, 100, 200, 300 when the true values of the parameters *α* = 0.6, λ = 0.9, *β* = 0.5, *b* = 2.

*n*	Parameters	Estimate	Average Bias	MSE	Lower Limit	Upper Limit	Length
50	*α*	0.7936	0.1936	0.0897	0.5059	1.0814	0.5755
	λ	1.1027	0.2027	0.0404	0.8196	1.3859	0.5663
	*β*	0.5542	0.0542	0.0099	0.4386	0.6698	0.2312
	*b*	2.0274	0.0274	0.0024	1.6853	2.3694	0.6841
100	*α*	0.7729	0.1729	0.0487	0.6172	0.9286	0.3114
	λ	1.0893	0.1893	0.0175	0.9436	1.2350	0.2914
	*β*	0.5371	0.0371	0.0061	0.4774	0.5968	0.1194
	*b*	2.0151	0.0151	0.0011	1.8209	2.2093	0.3884
200	*α*	0.7394	0.1394	0.0236	0.6724	0.8064	0.1340
	λ	1.0786	0.1786	0.0091	1.0008	1.1564	0.1556
	*β*	0.5234	0.0234	0.0047	0.4957	0.5511	0.0554
	*b*	2.0058	0.0058	0.0004	1.9129	2.0986	0.1857
300	*α*	0.7212	0.1212	0.0151	0.6903	0.7521	0.0618
	λ	1.0721	0.1721	0.0053	1.0358	1.1084	0.0726
	*β*	0.5112	0.0112	0.0032	0.4998	0.5226	0.0228
	*b*	2.0024	0.0024	0.0001	1.9645	2.0403	0.0758

For *b* = 2, the estimates tend to be larger, reflecting heavier tails. As with *b* = 1, the accuracy improves with larger *n*, as seen by reduced bias and MSE. The estimate for λ improves from 1.1027 at *n* = 50 to 1.0721 at *n* = 300.

#### 6.2.3 Coverage probability (Table 9)

**Table 9:** Coverage probability for sample sizes *n* = 50, 100, 200, 300 when *b* = 1 and *b* = 2.

*n*	*α* (*b* = 1)	λ (*b* = 1)	*α* (*b* = 2)	λ (*b* = 2)
50	0.90	0.88	0.84	0.83
100	0.93	0.92	0.88	0.87
200	0.95	0.94	0.92	0.91
300	0.96	0.95	0.94	0.93

The coverage probability approaches the nominal 95% as *n* increases. For *b* = 1, it reaches 0.96 at *n* = 300, while for *b* = 2, it reaches 0.93.

## 7 Application

In this section, we compare the performance of the TLEC distribution through a real life data with four other distributions, namely the Chen distribution that is proposed by [14], the exponentiated chen (EC) distribution by [20], the logistic Chen (LC) distribution by [22], the Topp Leone Chen (TLChen) distribution by [23] and the exponentially generated modify Chen distribution (EGMC) by [24]. More precisely, the values of the MLE parameters of the TLEC distribution are computed using neldermead package in R programming language.

The accuracy of the obtained estimates is evaluated using some measures such as Akaike information criteria (AIC), Bayesian information criteria (BIC), consistent Akaike information criteria (CAIC), and Hannan and quin information criteria (HQIC) (see [25]) A better fit to the data is achieved when the value of these statistics for the TLEC distribution is lower than other generalization of Chen distribution.

### 7.1 The infected cases of Saudi Arabia

This data include cases that were infected between 1 January and 6 March 2022 in Saudi Arabia. The observations of this dataset are presented in Table 10 which are available at https://covid19.moh.gov.sa.

**Table 10:** The infected data of Saudi Arabia for 65 days.

1024	1746	2585	3045	3168	3575	3068
3460	4778	4652	5362	5499	5628	5281
5477	5505	5873	5928	5591	4884	4608
4535	4838	4541	4526	4738	4474	3913
3669	4211	3861	4092	3852	3555	3013
3260	3747	3330	3162	2866	2523	1726
2136	2227	1982	1793	1569	1376	997
1031	1052	841	627	677	664	537
632	653	563	476	407	363	283
317	279					

The estimates for the new proposed distribution and four other related distributions are listed in Table 11. The log-likelihood, AIC, CAIC, BIC and HQIC for each model are summarized in Table 12. It can be observed that our new distribution has minimum values of all four criteria in comparison to the five distributions.

**Table 11:** Parameters estimation with their standard errors (in parenthesis) of the TLEC distribution and the four other existing distributions for the infected cases data.

Distribution	$\hat{\alpha }$	$\hat{\lambda }$	$\hat{\beta }$	$\hat{b}$
TLEC	0.5901	0.0001	0.2586	1.2118
	(0.1005)	(0.0001)	(0.0114)	(0.0582)
EGMC	1.0141	0.0079	0.2417	0.1009
	(0.8701)	(0.0252)	(0.1112)	(0.0294)
EC	⋯	0.0008	0.2416	1.0321
		(0.0001)	(0.0112)	(0.1627)
CHEN	⋯	0.0007	0.2435	⋯
		(0.0004)	(0.0087)	
TLChen	0.9032	0.0002	0.2474	⋯
	(0.0004)	(0.0001)	(0.0083)	
LC	1.4079	0.0035	0.2099	⋯
	(0.2425)	(0.0015)	(0.0106)	

**Table 12:** Goodness-of-fit test results of the TLEC distribution and the four other existing distributions for the infected cases data.

Distribution	-2logLik	AIC	CAIC	BIC	HQIC
TLEC	1146.58	1154.580	1167.278	1163.278	1158.012
EGMC	1148.74	1156.740	1169.438	1165.438	1160.172
EC	1148.84	1156.840	1169.538	1165.538	1160.272
CHEN	1148.64	1156.640	1169.338	1165.338	1160.272
TLChen	1148.06	1156.060	1168.758	1164.758	1159.492
LC	1154.70	1167.492	1180.190	1176.190	1170.924

**Fig 3:**
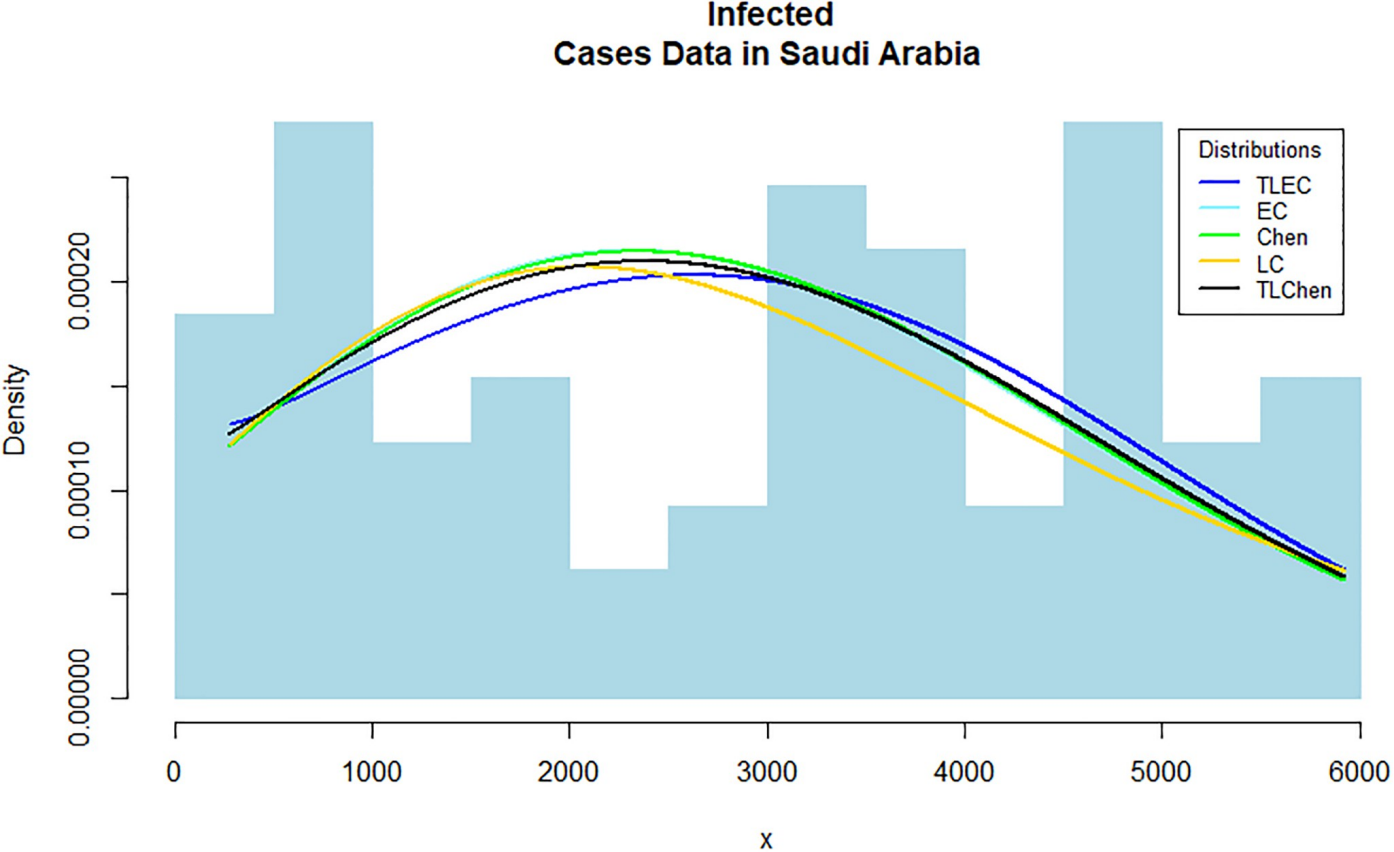
Estimated densities for the infected cases data in Saudi Arabia.

**Fig 4:**
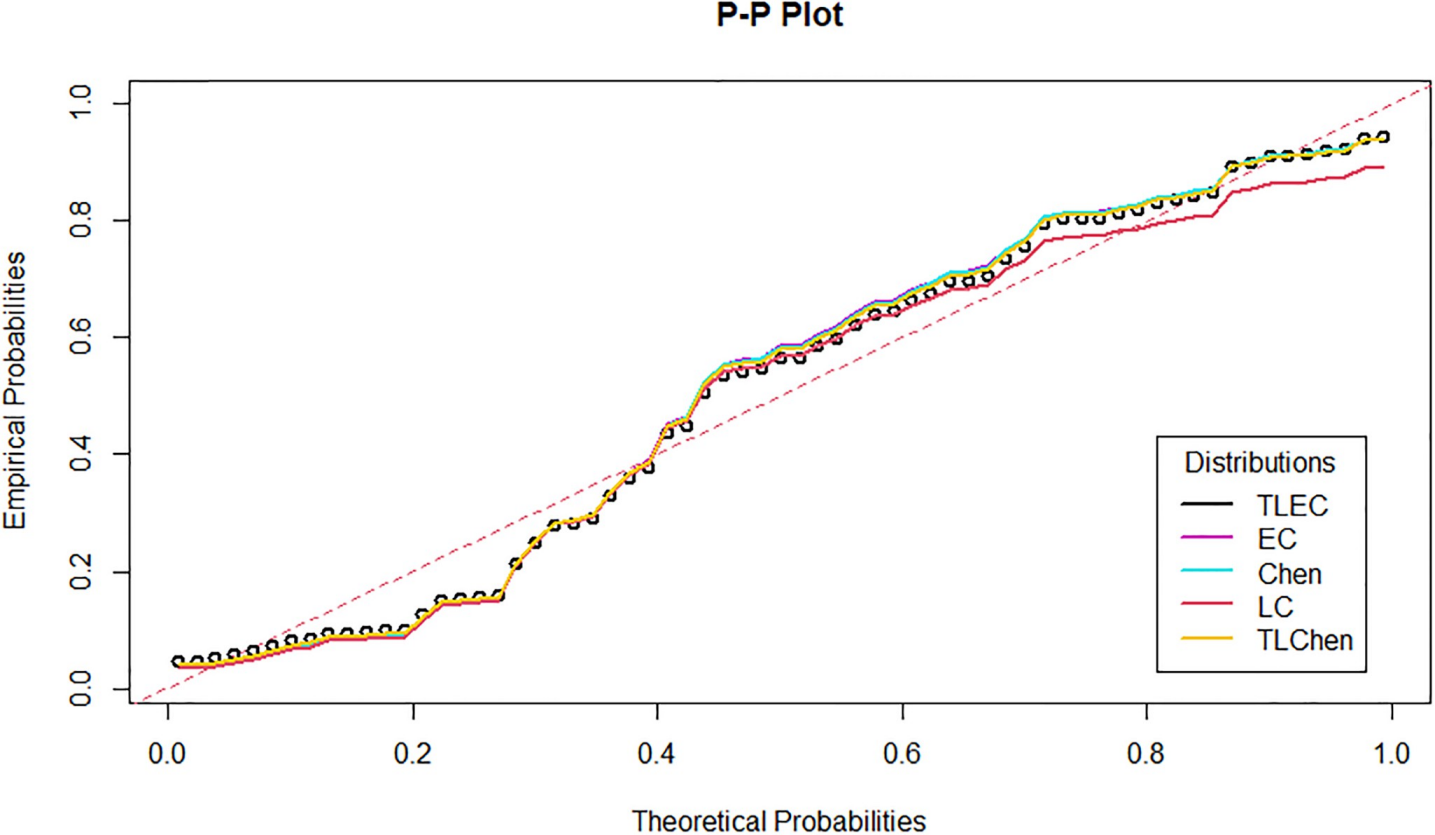
P-P plot for the infected cases data in Saudi Arabia.

**Fig 5:**
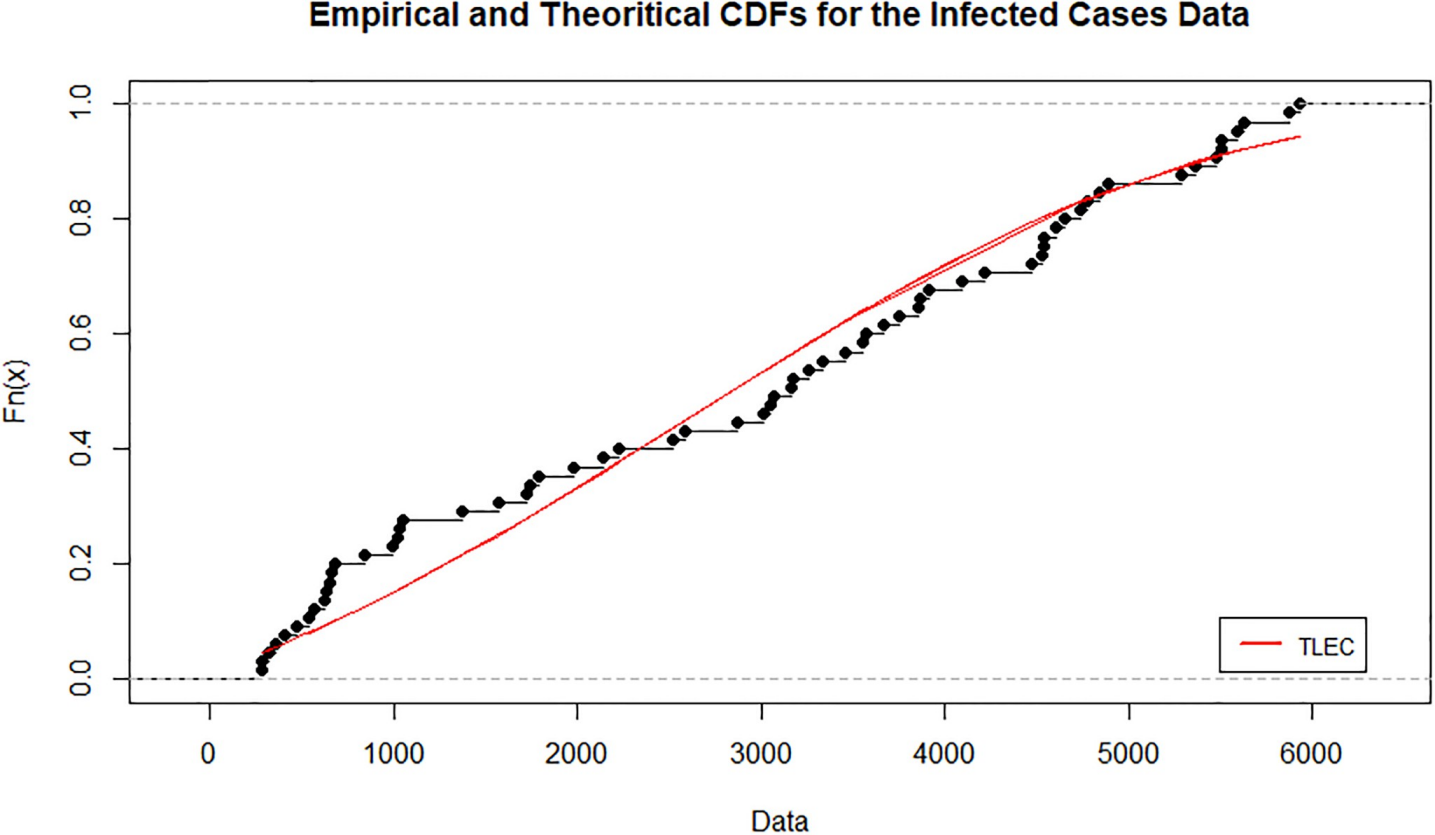
Empirical and Theoritical CDFs for the infected cases data in Saudi Arabia.

**Fig 6:**
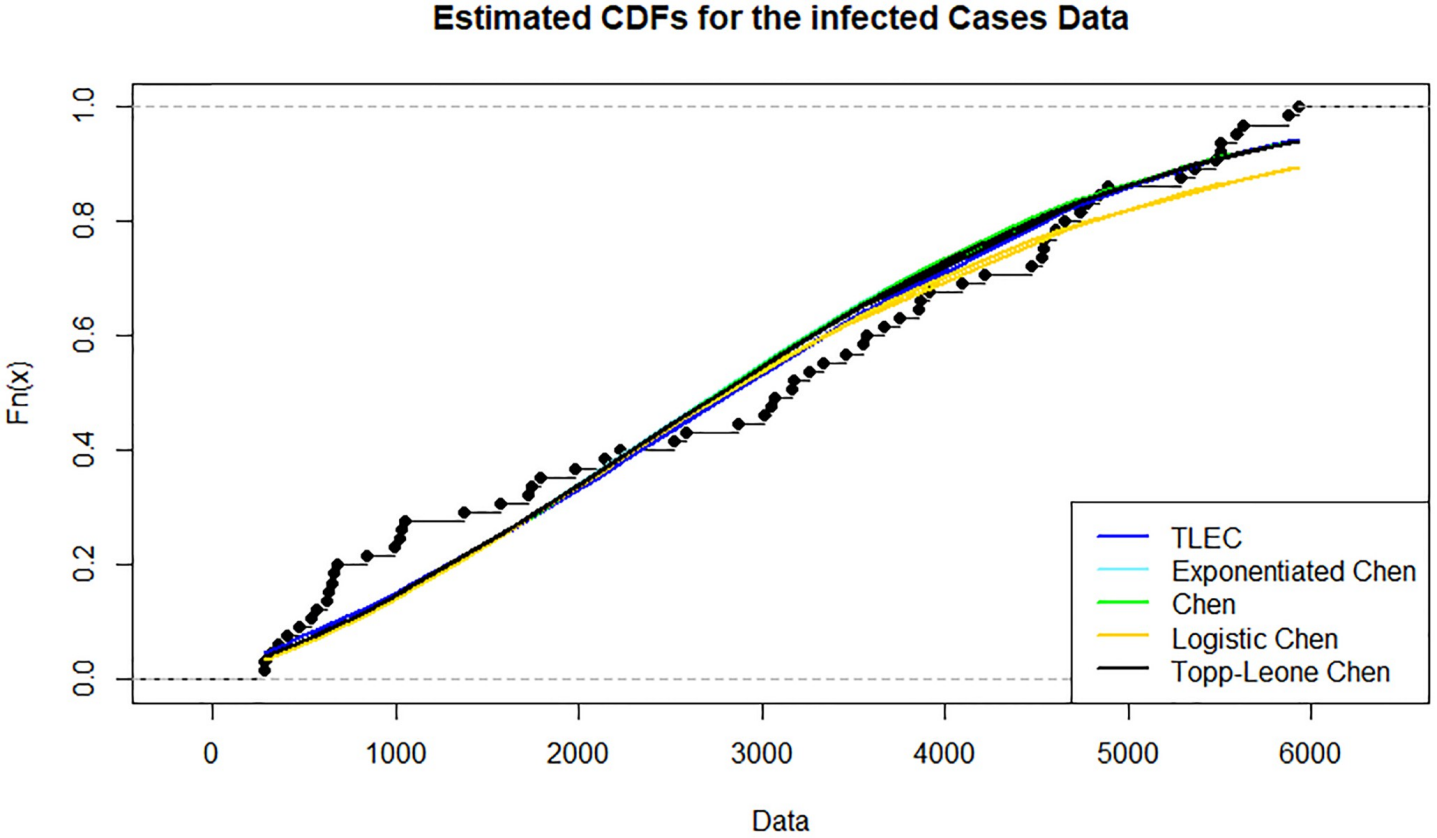
Estimated CDFs for the infected cases data in Saudi Arabia.

**Fig 7:**
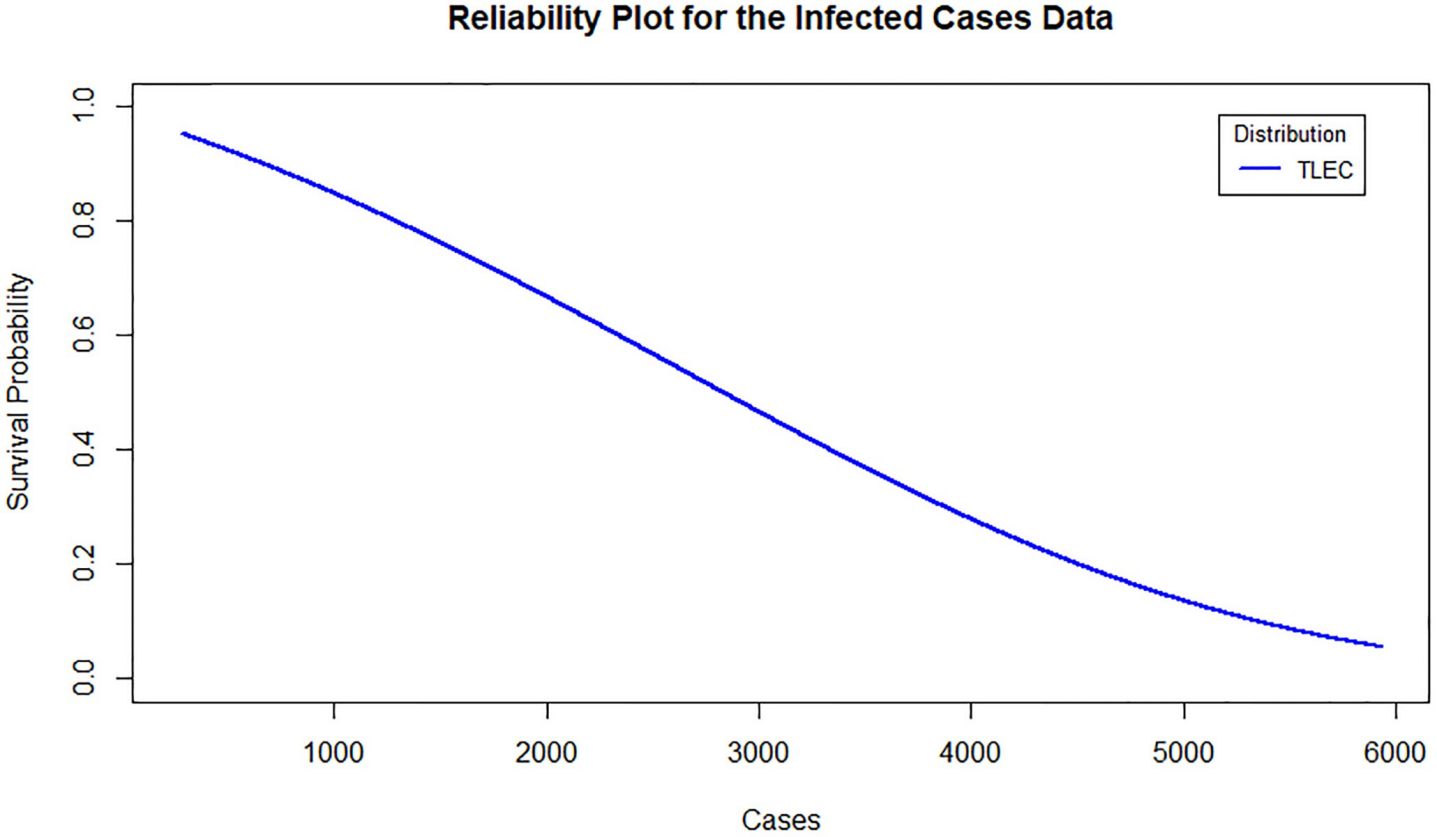
Estimated reliability density for the infected cases data in Saudi Arabia.

Fig 3 shows the estimated density functions for the TLEC and other distributions. The TLEC distribution clearly fits the data well, with a more pronounced curve that follows the central data points closely compared to the other distributions. This highlights the flexibility and accuracy of the TLEC distribution in modeling the infected cases data. The Probability-probability plot (PP plot) in Fig 4 illustrates how well the sample data fits our proposed distribution in relation to other distributions. Figs 5 and 6 illustrate the empirical and theoretical cumulative distribution functions (CDFs) for the infected cases data. The close alignment between the empirical data points and the TLEC’s theoretical CDF underscores the accuracy of the TLEC model in capturing the overall distribution of the data. Fig 7 depicts the reliability (survival) function for the TLEC distribution. The smooth decrease in survival probability over time indicates the model’s ability to track the likelihood of cases with high precision, showing a consistent decline as cases increase.

### 7.2 The recovered cases of Saudi Arabia

The following data is about the recovered cases that were occurred between 1 January and 6 March 2022 in Saudi Arabia. The information is available at https://covid19.moh.gov.sa. The data are shown in Table 13.

**Table 13:** The recovered data of Saudi Arabia for 65 days.

298	341	375	424	608	817	793
843	893	2051	2499	2978	3511	2996
3405	4349	4535	4981	5238	6090	4622
5072	6296	5212	5772	4973	4445	4284
4375	5162	4377	4604	4638	4023	4824
3878	4083	3464	4088	3379	3825	2983
2983	3469	3372	3207	2847	2596	1928
2136	2036	1922	1880	1585	1409	1085
995	1081	839	719	685	559	525
668	645					

Table 14 displays the estimated parameters of the TLEC distribution and other associated distributions. By looking at Table 15, it is clear that the TLEC distribution performs better than the other distributions since it has the lowest AIC, CAIC, BIC, and HQIC values.

**Table 14:** Parameters estimation with their standard errors (in parenthesis) of the TLEC distribution and the four other existing distributions for the recovered cases data.

Distribution	$\hat{\alpha }$	$\hat{\lambda }$	$\hat{\beta }$	$\hat{b}$
TLEC	1.9779	0.0001	0.2561	0.4562
	(0.0137)	(0.0001)	(0.0109)	(0.0801)
EGMC	0.9629	0.0039	0.2446	0.1780
	(0.1586)	(0.0505)	(0.0244)	(0.0004)
EC	⋯	0.0035	0.2215	1.5732
		(0.0001)	(0.0106)	(0.1447)
CHEN	⋯	0.0007	0.2449	⋯
		(0.0003)	(0.0087)	
TLChen	2.4041	0.0054	0.2022	⋯
	(0.0006)	(0.0003)	(0.0091)	
LC	1.0493	0.0008	0.2423	⋯
	(0.1749)	(0.0004)	(0.0124)	

**Table 15:** Goodness-of-fit test results of the TLEC distribution and the four other existing distributions for the recovered cases data.

Distribution	-2logLik	AIC	CAIC	BIC	HQIC
TLEC	1144.32	1152.320	1165.018	1161.018	1155.752
EGMC	1144.36	1152.360	1165.058	1161.058	1155.792
EC	1146.64	1154.640	1167.338	1163.338	1158.072
CHEN	1144.44	1152.440	1165.138	1161.138	1155.872
TLChen	1148.84	1156.840	1169.538	1165.538	1160.272
LC	1145.28	1153.280	1165.978	1161.978	1156.712

**Fig 8:**
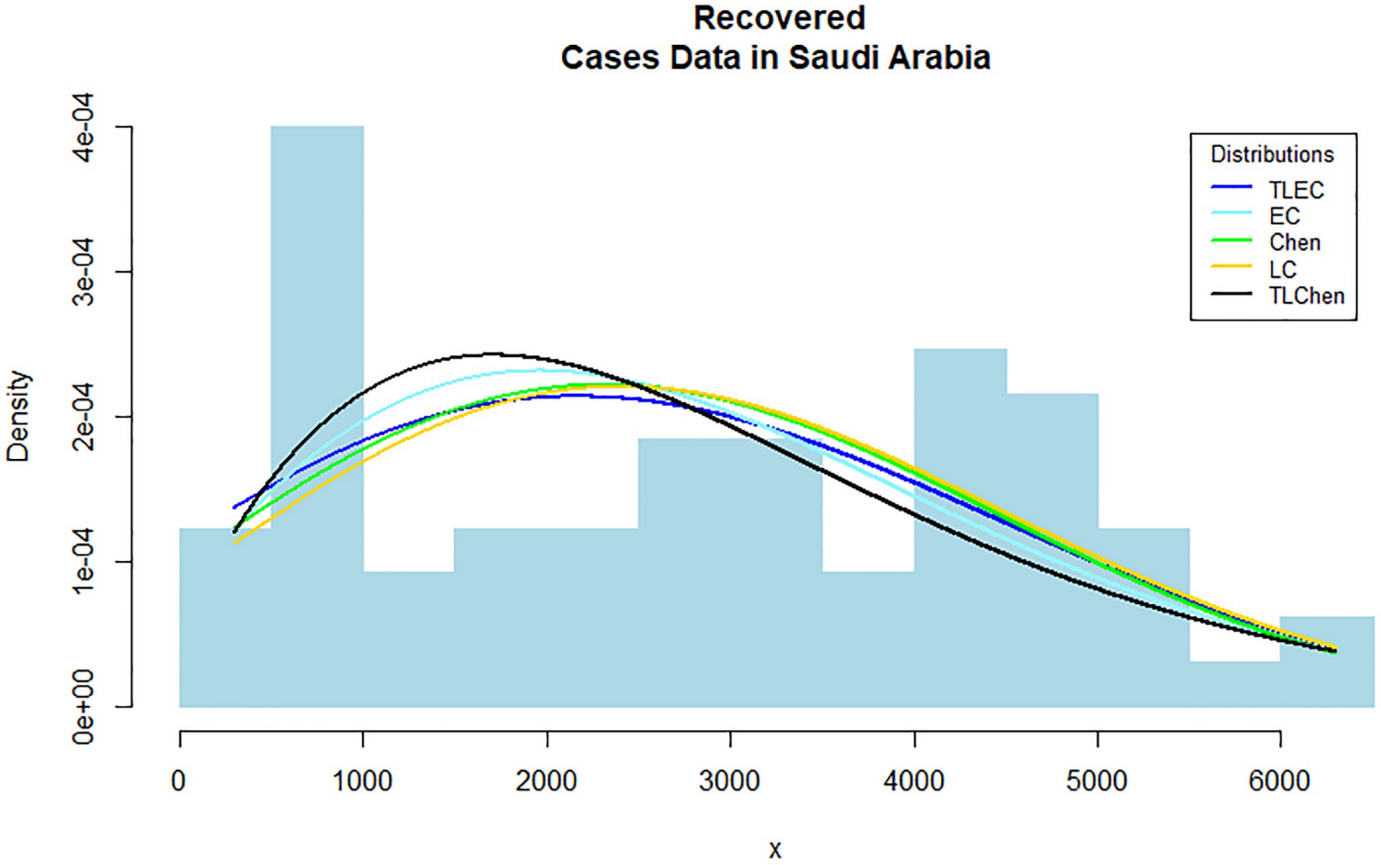
Estimated densities for the recovered cases data in Saudi Arabia.

**Fig 9:**
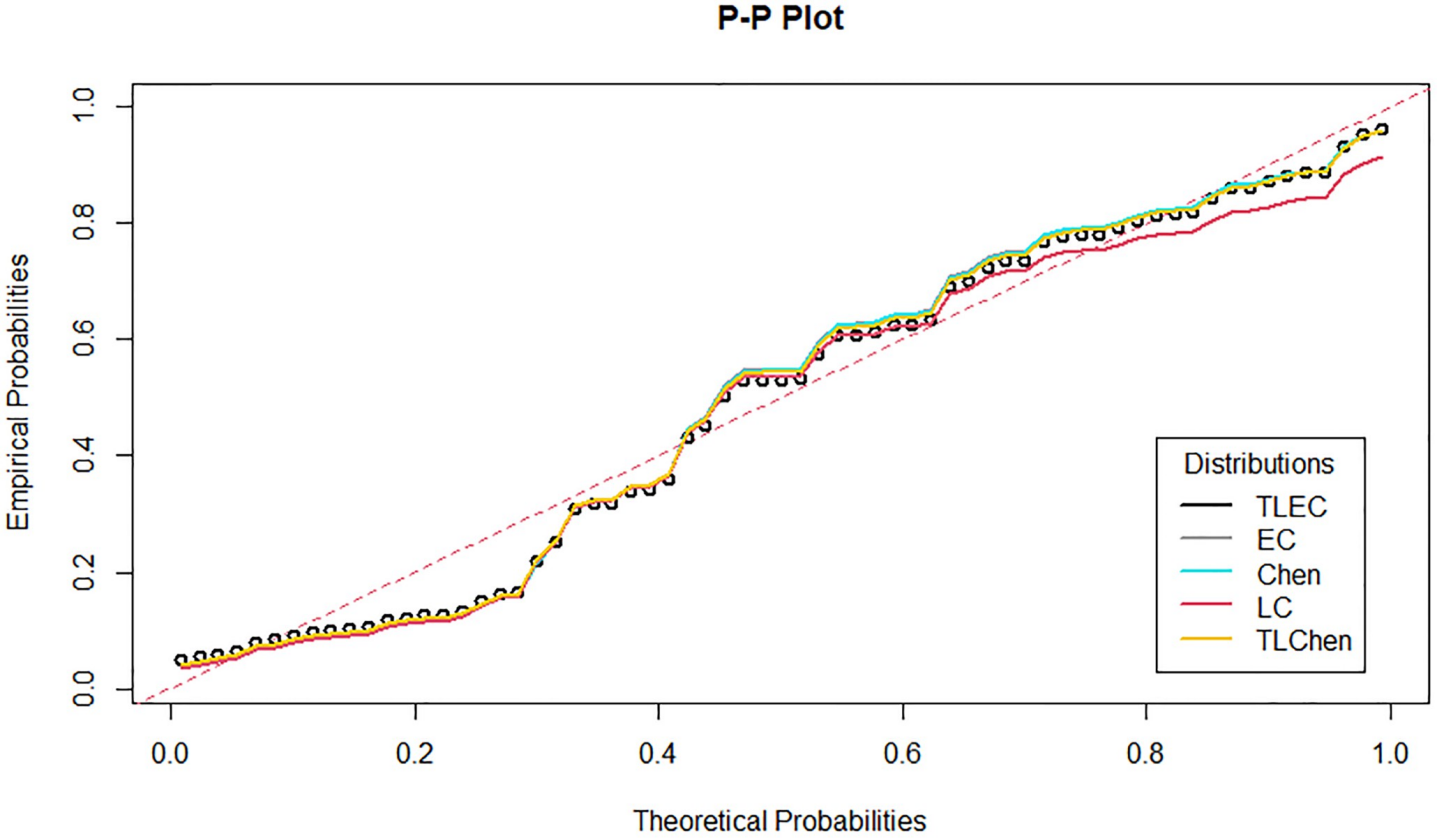
P-P plot for the recovered cases data in Saudi Arabia.

**Fig 10:**
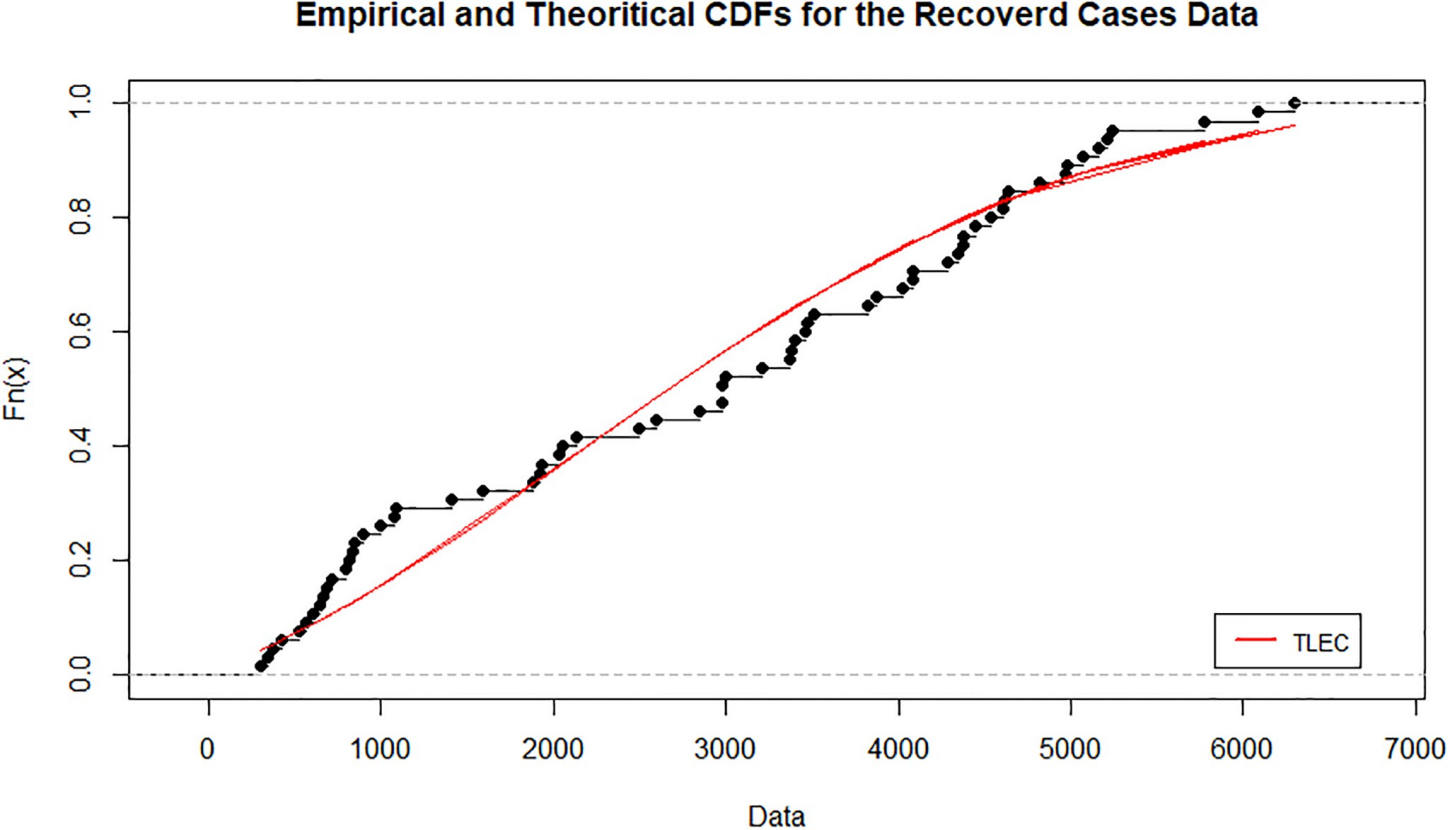
Empirical and Theoritical CDFs for the recovered cases data in Saudi Arabia.

**Fig 11:**
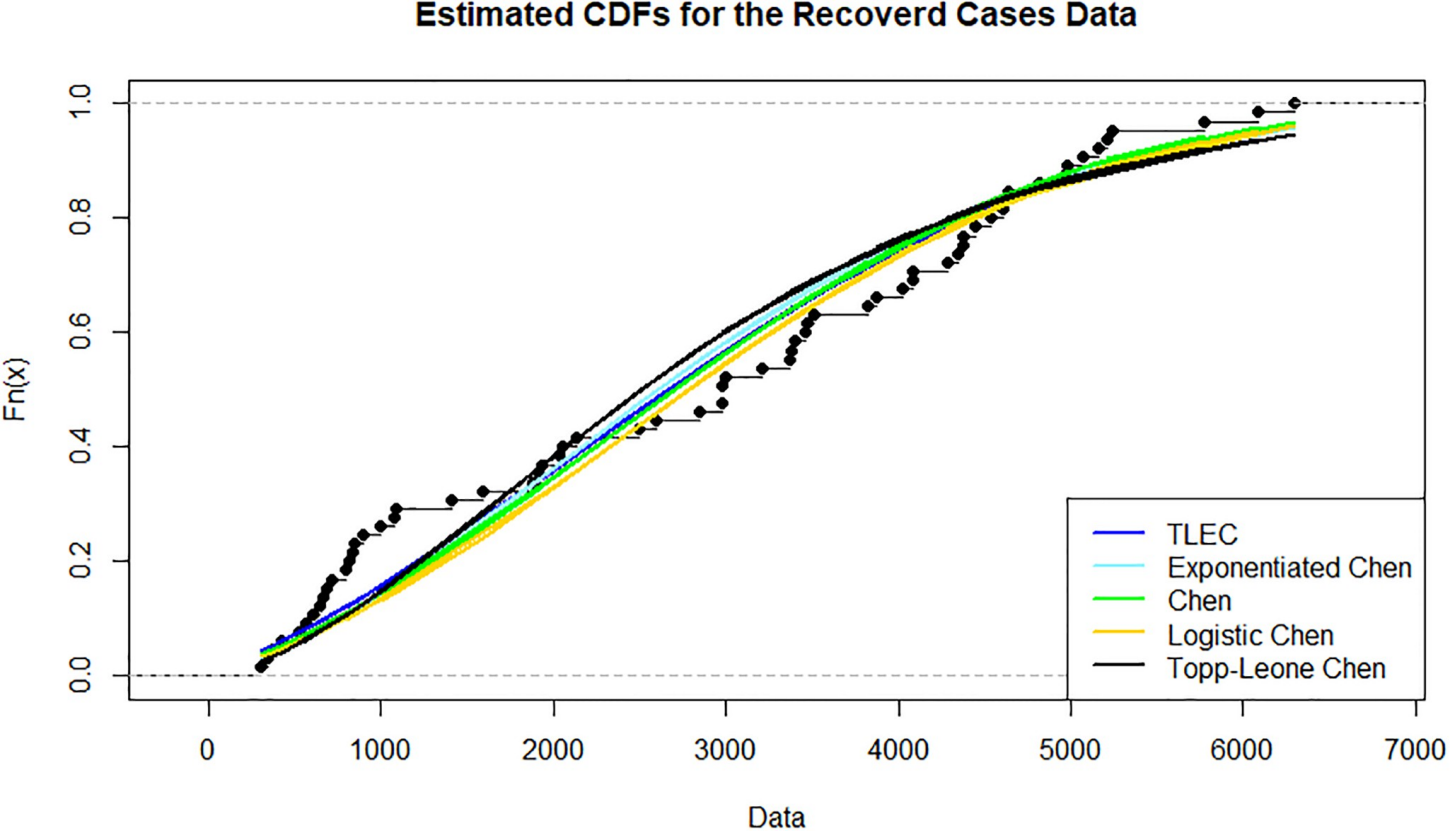
Estimated CDFs for the recovered cases data.

Fig 8 shows the estimated densities for the recovered cases data. The TLEC distribution provides a superior fit compared to other distributions, as it closely follows the observed data. It demonstrates its ability to capture the overall shape and variability of the dataset, highlighting its flexibility in fitting real-world data. The sample data’s suitability for our suggested distribution in comparison to other distributions can be observed by the P-P plot in Figs 9–11 present the empirical and CDFs for the recovered cases data. The TLEC distribution performs well, as the theoretical curve aligns closely with the empirical data points, confirming the good fit of the model.

**Fig 12:**
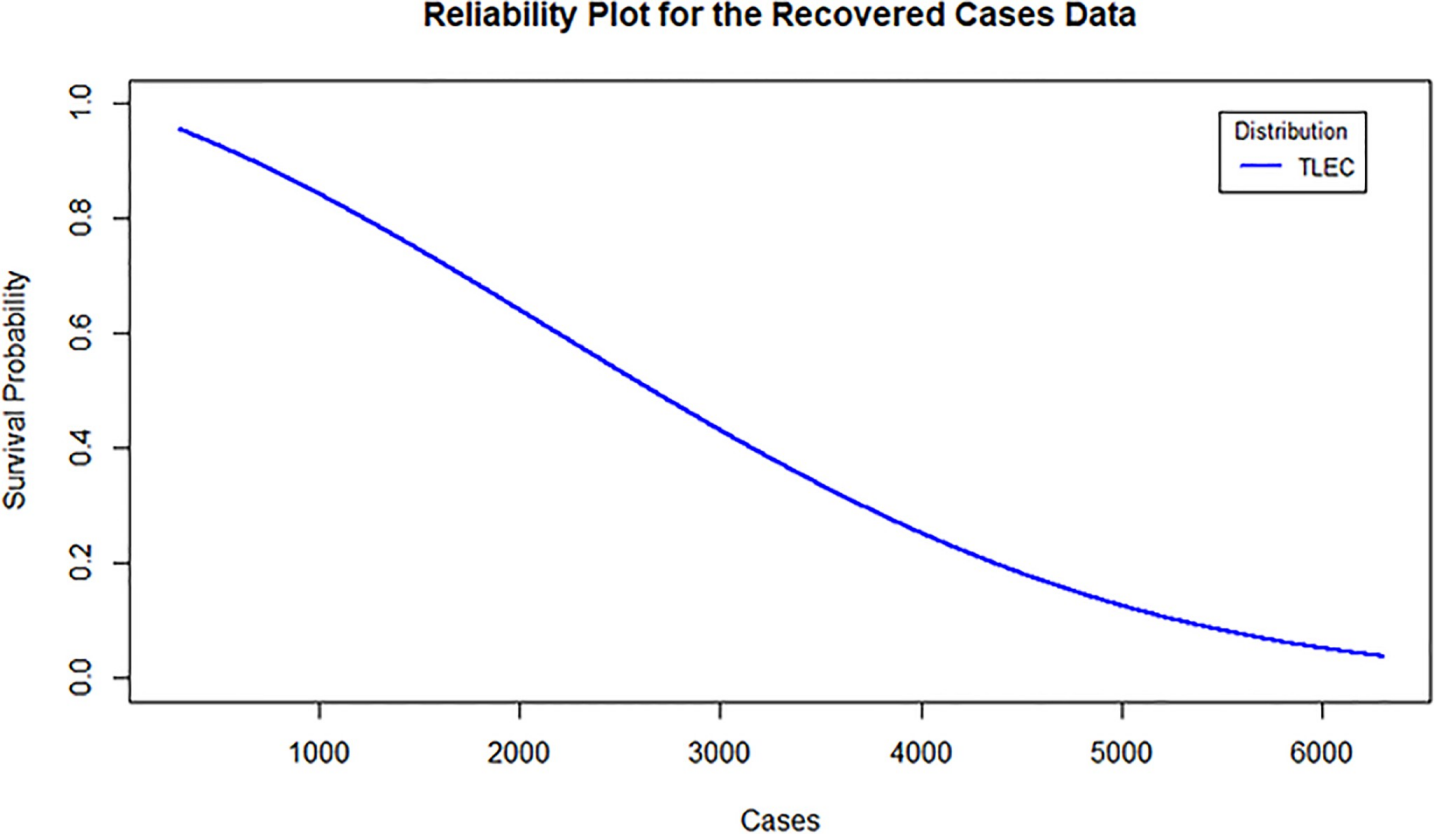
Estimated reliability density for the recovered cases data in Saudi Arabia.

Finally, Fig 12 displays the reliability plot for the recovered cases data, showcasing the survival probability over time. The TLEC distribution effectively captures the decreasing trend of survival probability, further demonstrating its applicability in real-life scenarios like recovery rates.

Together, these figures illustrate the robustness and flexibility of the TLEC distribution in modeling the recovered cases data in Saudi Arabia.

## 8 Discussion

The Topp-Leone Exponentiated Chen (TLEC) distribution provides a crucial advancement in the field of statistical modeling by offering a more flexible framework than many existing distributions. The **importance** of the TLEC distribution lies in its ability to model a wide variety of hazard rate shapes, including increasing, decreasing, and bathtub forms. This makes it particularly valuable in fields such as survival analysis, reliability engineering, and public health, where hazard rate flexibility is critical for accurate risk modeling.

Compared to the standard Chen and exponentiated Chen distributions, which are limited in their ability to capture certain hazard rate shapes, the TLEC distribution introduces additional shape parameters that allow for more comprehensive modeling of real-world phenomena. This added flexibility is of significant theoretical importance, as it allows the TLEC distribution to model a wider range of datasets and underlying processes that exhibit non-standard hazard rate behaviors.

From a **methodological perspective**, the TLEC distribution contributes to the existing literature by providing a new tool for data analysts and researchers who require models that can adapt to the diverse nature of real-world data. The derivation of key statistical properties, such as moments, order statistics, and the hazard rate function, further underscores the theoretical depth of this new distribution. Additionally, the application of the MLE method for parameter estimation has shown to be both efficient and reliable in various scenarios, as demonstrated by the simulation studies in this paper.

By applying the TLEC distribution to COVID-19 data, we demonstrate its **practical significance**. The TLEC distribution outperforms existing models, such as the Topp-Leone Chen and Logistic Chen distributions, in terms of goodness-of-fit metrics. This provides strong evidence that the TLEC distribution is not just a theoretical extension but a **practically useful tool** for real-world applications, particularly in public health contexts where accurate modeling of infection and recovery rates is essential.

Overall, the TLEC distribution represents an important step forward in statistical modeling. Its ability to capture complex hazard rate behaviors, combined with its practical utility in fitting real-world data, makes it a valuable addition to the statistical toolkit for researchers and practitioners alike. Future work will focus on expanding the TLEC distribution to multivariate settings, as well as exploring its applications in other domains such as biomedical sciences and engineering.

## 9 Conclusions

In this study, we have introduced the Topp-Leone Exponentiated Chen (TLEC) distribution, a new extended distribution that generalizes the Chen and exponentiated Chen distributions by incorporating the Topp-Leone generator. This distribution offers enhanced flexibility, particularly in capturing a wide variety of hazard rate shapes such as increasing, decreasing, and bathtub forms, which are common in survival analysis and reliability studies.

The key contributions of this work include the derivation of important statistical properties such as the probability density function, cumulative distribution function, moments, hazard rate function, and order statistics. Maximum likelihood estimation was employed for parameter estimation, and the efficiency of this method was demonstrated through simulation studies. The findings showed that as the sample size increases, the MLE becomes more accurate, further validating the applicability of the TLEC distribution.

When applied to real COVID-19 data from Saudi Arabia, the TLEC distribution outperformed related models, such as the Chen, Topp-Leone Chen, and Logistic Chen distributions, providing superior goodness-of-fit metrics (AIC, CAIC, BIC and HQIC). This highlights the distribution’s practical utility for modeling real-world phenomena with varying hazard rate behaviors.

Despite the promising results, the current study has some limitations. First, the TLEC distribution was only applied to univariate data. Its performance in multivariate or bivariate contexts, which are important for modeling dependent structures in data, remains unexplored. Additionally, while maximum likelihood estimation was effective, alternative parameter estimation methods such as Bayesian inference or robust estimators were not explored. Lastly, the model was tested on a single dataset (COVID-19 cases), and further validation across various types of real-world data is necessary to confirm its generalizability.

The study presents a significant improvement in modeling flexibility through the TLEC distribution, particularly in its ability to model various hazard rate shapes, which are essential for a wide range of applications in survival analysis and reliability engineering. The superior performance of the TLEC distribution in fitting COVID-19 data suggests it can be a valuable tool in public health and other domains requiring accurate risk estimation and prediction.

We suggest further research to compare the TLEC distribution with other emerging flexible distributions across different real-world datasets to assess its relative strengths and weaknesses. Additionally, the potential for the TLEC distribution to model other types of complex data, such as time series or spatial data, warrants further investigation.

Several avenues for future research are planned to build on the findings of this study. First, we intend to extend the TLEC distribution to multivariate and bivariate settings to capture dependencies between variables. This will be crucial for applications in areas such as finance, weather forecasting, and biology, where multiple variables are often interdependent. Second, exploring Bayesian inference methods for parameter estimation could provide more robust estimates, particularly in the presence of small sample sizes or outliers. Lastly, the TLEC distribution will be tested on diverse real-world datasets from fields like engineering, biomedical sciences, and economics, to further validate its versatility and robustness in different contexts.

## Supporting information

S1 Appendix (ZIP)

S1 Data (XLSX)

S2 Data (XLSX)
