# Supplementary material for: A new extended Chen distribution for modelling COVID-19 data
Source: PLoS One. 2025 Jan 3;20(1):e0316235. doi: 10.1371/journal.pone.0316235 (PMC11698366; doi:10.1371/journal.pone.0316235)
Supplement: S1 Appendix — (ZIP) [file pone.0316235.s001.zip › main.pdf]

Appendix

The observed information matrix can be written as follows:

$$I(\Theta) = -\begin{bmatrix} \frac{\partial^2\ell}{\partial\alpha^2} & \frac{\partial^2\ell}{\partial\alpha\partial\beta} & \frac{\partial^2\ell}{\partial\alpha\partial\lambda} & \frac{\partial^2\ell}{\partial\alpha\partial b} \\ & \frac{\partial^2\ell}{\partial\beta^2} & \frac{\partial^2\ell}{\partial\beta\partial\lambda} & \frac{\partial^2\ell}{\partial\beta\partial b} \\ & & \frac{\partial^2\ell}{\partial\lambda^2} & \frac{\partial^2\ell}{\partial\lambda\partial b} \\ & & & \frac{\partial^2\ell}{\partial b^2} \end{bmatrix}$$

where $\Theta = (\alpha, \beta, \lambda, b)^{'}$. The elements of the matrix are given below:

$$\frac{\partial^2\ell}{\partial\alpha^2} = \frac{-n}{\alpha^2} \tag{1}$$

$$\frac{\partial^2\ell}{\partial\alpha\partial\beta} = \frac{2\left(1-e^{\lambda\left(1-e^{x_i^\beta}\right)}\right)^{b-1} b e^{\lambda\left(1-e^{x_i^\beta}\right)} \lambda e^{x_i^\beta} x_i \left(1-\left(1-e^{\lambda\left(1-e^{x_i^\beta}\right)}\right)^b\right)}{1-\left(1-\left(1-e^{\lambda\left(1-e^{x_i^\beta}\right)}\right)^b\right)^2}, \tag{2}$$

$$\frac{\partial^2\ell}{\partial\alpha\partial\lambda} = -\frac{2\left(1-e^{\lambda\left(1-e^{x_i^\beta}\right)}\right)^{b-1} b e^{\lambda\left(1-e^{x_i^\beta}\right)} \left(1-e^{x_i^\beta}\right) \left(1-\left(1-e^{\lambda\left(1-e^{x_i^\beta}\right)}\right)^b\right)}{1-\left(1-\left(1-e^{\lambda\left(1-e^{x_i^\beta}\right)}\right)^b\right)^2}, \tag{3}$$

$$\frac{\partial^2\ell}{\partial\alpha\partial b} = \frac{2\left(1-e^{\lambda\left(1-e^{x_i^\beta}\right)}\right)^{b} \log\left(1-e^{\lambda\left(1-e^{x_i^\beta}\right)}\right) \left(1-\left(1-e^{\lambda\left(1-e^{x_i^\beta}\right)}\right)^b\right)}{1-\left(1-\left(1-e^{\lambda\left(1-e^{x_i^\beta}\right)}\right)^b\right)^2}, \tag{4}$$

$$
\begin{aligned}
\frac{\partial^2 \ell}{\partial \beta^2} = {} & \sum_{i=1}^{n} x_i^{\beta} \log(x_i)^2 - \frac{n}{\beta^2} - \lambda \sum_{i=1}^{n} 2 x_i e^{x_i^{\beta}} \\
& + (b-1) \sum_{i=1}^{n} \left(\frac{2 x_i e^{\lambda\left(1-e^{x_i^{\beta}}\right)} \lambda e^{x_i^{\beta}} - e^{\lambda\left(1-e^{x_i^{\beta}}\right)} \left(\lambda e^{x_i^{\beta}} x_i\right)^2}{1 - e^{\lambda\left(1-e^{x_i^{\beta}}\right)}} \right) - \sum_{i=1}^{n} \frac{\left(e^{\lambda\left(1-e^{x_i^{\beta}}\right)} \lambda e^{x_i^{\beta}} x_i\right)^2}{\left(1 - e^{\lambda\left(1-e^{x_i^{\beta}}\right)}\right)^2} \\
& - \sum_{i=1}^{n} \left(\frac{\lambda b e^{\lambda\left(1-e\, x_i{}^{\beta\, b}\right)} \, (\left(\left(1-e^{x_i{}^{\beta}}\right)^{b-1} e^{x_i^{\beta}} x_i^2 - b\,(b-1) \left(1-e^{x_i^{\beta}}\right)^{b-2} (e^{x_i^{\beta}})^2 x_i^2\right)}{1 - \left(1 - e^{\lambda\left(1-e^{x_i^{\beta\, b}}\right)}\right)} \right) \\
& - \sum_{i=1}^{n} \frac{b e^{\lambda\left(1-e^{x_i^{\beta\, b}}\right)} \left(\lambda \left(1-e^{x_i^{\beta}}\right)^{b-1} e^{x_i^{\beta}} x_i\right)^2}{1 - \left(1 - e^{\lambda\left(1-e^{x_i^{\beta\, b}}\right)}\right)} + \sum_{i=1}^{n} \frac{\left(e^{\lambda\left(1-e^{x_i^{\beta\, b}}\right)} \lambda \left(1-e^{x_i^{\beta}}\right)^{b-1} b e^{x_i^{\beta}} x_i\right)^2}{\left(1 - \left(1 - e^{\lambda\left(1-e^{x_i^{\beta\, b}}\right)}\right)\right)^2}) \\
& + (\alpha-1) \sum_{i=1}^{n} \left(\frac{2 \left(\left(1 - e^{\lambda\left(1-e^{x_i^{\beta}}\right)}\right)^{b-2} (b-1)\, e^{\lambda\left(1-e^{x_i^{\beta}}\right)} \lambda e^{x_i^{\beta}} x_i b e^{\lambda\left(1-e^{x_i^{\beta}}\right)} \lambda e^{x_i^{\beta}} x_i \right)}{1 - \left(1 - \left(1 - e^{\lambda\left(1-e^{x_i^{\beta}}\right)}\right)^{b}\right)^2} \right) \\
& + \sum_{i=1}^{n} \frac{\left(1 - e^{\lambda\left(1-e^{x_i^{\beta}}\right)}\right)^{b-1} b(e^{\lambda\left(1-e^{x_i^{\beta}}\right)} \lambda e^{x_i^{\beta}} x_i^2}{1 - \left(1 - \left(1 - e^{\lambda\left(1-e^{x_i^{\beta}}\right)}\right)^{b}\right)^2} \\
& - \sum_{i=1}^{n} \frac{e^{\lambda\left(1-e^{x_i^{\beta}}\right)} \left(\lambda e^{x_i^{\beta}} x_i\right)^2 \left(1 - \left(1 - e^{\lambda\left(1-e^{x_i^{\beta}}\right)}\right)^{b}\right)}{1 - (1 - (1 - e^{\lambda\left(1-e^{x_i^{\beta}}\right)})^{b})^2} - \sum_{i=1}^{n} \frac{\left(\left(1 - e^{\lambda\left(1-e^{x_i^{\beta}}\right)}\right)^{b-1} b e^{\lambda\left(1-e^{x_i^{\beta}}\right)} \lambda e^{x_i^{\beta}} x_i\right)^2}{1 - \left(1 - \left(1 - e^{\lambda\left(1-e^{x_i^{\beta}}\right)}\right)^{b}\right)^2} \\
& - 4 \sum_{i=1}^{n} \frac{\left(\left(1 - e^{\lambda\left(1-e^{x_i^{\beta}}\right)}\right)^{b-1} b e^{\lambda\left(1-e^{x_i^{\beta}}\right)} \lambda e^{x_i^{\beta}} x_i \left(1 - \left(1 - e^{\lambda\left(1-e^{x_i^{\beta}}\right)}\right)^{b}\right)\right)^2}{\left(1 - \left(1 - \left(1 - e^{\lambda\left(1-e^{x_i^{\beta}}\right)}\right)^{b}\right)^2\right)^2},
\end{aligned}
\tag{5}
$$

$$\frac{\partial^2 \ell}{\partial\beta\partial\alpha}=\sum_{i=1}^{n}\frac{2\left(1-e^{\lambda\left(1-e^{x_i^\beta}\right)}\right)^{b-1}be^{\lambda\left(1-e^{x_i^\beta}\right)}\lambda e^{x_i^\beta}x_i\left(1-\left(1-e^{\lambda\left(1-e^{x_i^\beta}\right)}\right)^b\right)}{1-\left(1-\left(1-e^{\lambda\left(1-e^{x_i^\beta}\right)}\right)^b\right)^2}, \tag{6}$$

$$\begin{aligned}\frac{\partial^2 \ell}{\partial\beta\partial\lambda}=&-\sum_{i=1}^{n}e^{x_i^\beta}x_i+(b-1)\sum_{i=1}^{n}\frac{e^{\lambda\left(1-e^{x_i^\beta}\right)}\left(1-e^{x_i^\beta}\right)\lambda e^{x_i^\beta}x_i+e^{\lambda\left(1-e^{x_i^\beta}\right)}e^{x_i^\beta}x_i}{1-e^{\lambda\left(1-e^{x_i^\beta}\right)}}\\&+\sum_{i=1}^{n}\frac{e^{\lambda\left(1-e^{x_i^\beta}\right)}\lambda e^{x_i^\beta}x_ie^{\lambda\left(1-e^{x_i^\beta}\right)}\left(1-e^{x_i^\beta}\right)}{\left(1-e^{\lambda\left(1-e^{x_i^\beta}\right)}\right)^2}\\&-\sum_{i=1}^{n}\frac{e^{\lambda\left(1-e^{x_i^\beta}\right)^b}\left(1-e^{x_i^\beta}\right)^b\lambda\left(1-e^{x_i^\beta}\right)^{b-1}be^{x_i^\beta}x_i+e^{\lambda\left(1-e^{x_i^\beta}\right)^b}\left(1-e^{x_i^\beta}\right)^{b-1}be^{x_i^\beta}x_i}{1-\left(1-e^{\lambda\left(1-e^{x_i^\beta}\right)^b}\right)}\\&-\sum_{i=1}^{n}\frac{e^{\lambda\left(1-e^{x_i^\beta}\right)^b}\lambda\left(1-e^{x_i^\beta}\right)^{b-1}be^{x_i^\beta}x_ie^{\lambda\left(1-e^{x_i^\beta}\right)^b}\left(1-e^{x_i^\beta}\right)^b}{\left(1-\left(1-e^{\lambda\left(1-e^{x_i^\beta}\right)^b}\right)\right)^2}\\&+(\alpha-1)\sum_{i=1}^{n}\frac{2\left(1-e^{\lambda\left(1-e^{x_i^\beta}\right)}\right)^{b-1}b\left(e^{\lambda\left(1-e^{x_i^\beta}\right)}\left(1-e^{x_i^\beta}\right)\lambda e^{x_i^\beta}x_i\right)}{1-\left(1-\left(1-e^{\lambda\left(1-e^{x_i^\beta}\right)}\right)^b\right)^2},\end{aligned} \tag{7}$$

$$
\begin{aligned}
\frac{\partial^2 \ell}{\partial \beta \partial b} = & \sum_{i=1}^{n} \frac{e^{\lambda\left(1-e^{x_i^{\beta}}\right)} \lambda e^{x_i^{\beta}} x_i}{1-e^{\lambda\left(1-e^{x_i^{\beta}}\right)}} \\
& - \sum_{i=1}^{n} \frac{e^{\lambda\left(1-e^{x_i^{\beta}}\right)^{b}} \lambda \left(1-e^{x_i^{\beta}}\right)^{b} \log\left(1-e^{x_i^{\beta}}\right) \lambda \left(1-e^{x_i^{\beta}}\right)^{b-1} b e^{x_i^{\beta}} x_i}{1-\left(1-e^{\lambda\left(1-e^{x_i^{\beta}}\right)^{b}}\right)} \\
& + \sum_{i=1}^{n} \frac{e^{\lambda\left(1-e^{x_i^{\beta}}\right)^{b}} \lambda \left(\left(1-e^{x_i^{\beta}}\right)^{b-1} \log\left(1-e^{x_i^{\beta}}\right) b e^{x_i^{\beta}} x_i + \left(1-e^{x_i^{\beta}}\right)^{b-1} e^{x_i^{\beta}} x_i\right)}{1-\left(1-e^{\lambda\left(1-e^{x_i^{\beta}}\right)^{b}}\right)} \\
& - \sum_{i=1}^{n} \frac{e^{\lambda\left(1-e^{x_i^{\beta}}\right)^{b}} \lambda \left(1-e^{x_i^{\beta}}\right)^{b-1} b e^{x_i^{\beta}} x_i e^{\lambda\left(1-e^{x_i^{\beta}}\right)^{b}} \lambda \left(1-e^{x_i^{\beta}}\right)^{b} \log\left(1-e^{x_i^{\beta}}\right)}{\left(1-\left(1-e^{\lambda\left(1-e^{x_i^{\beta}}\right)^{b}}\right)\right)^{2}} \\
& + (\alpha-1) \sum_{i=1}^{n} \frac{2\left(1-e^{\lambda\left(1-e^{x_i^{\beta}}\right)}\right)^{b-1} \log\left(1-e^{\lambda\left(1-e^{x_i^{\beta}}\right)}\right) b e^{\lambda\left(1-e^{x_i^{\beta}}\right)} \lambda e^{x_i^{\beta}} x_i}{1-\left(1-\left(1-e^{\lambda\left(1-e^{x_i^{\beta}}\right)}\right)^{b}\right)^{2}} \\
& + \sum_{i=1}^{n} \frac{\left(1-e^{\lambda\left(1-e^{x_i^{\beta}}\right)}\right)^{b-1} e^{\lambda\left(1-e^{x_i^{\beta}}\right)} \lambda e^{x_i^{\beta}} \log\left(e^{x_i}\right) \left(1-\left(1-e^{\lambda\left(1-e^{x_i^{\beta}}\right)}\right)^{b}\right)}{1-\left(1-\left(1-e^{\lambda\left(1-e^{x_i^{\beta}}\right)}\right)^{b}\right)^{2}} \\
& - \sum_{i=1}^{n} \frac{\left(1-e^{\lambda\left(1-e^{x_i^{\beta}}\right)}\right)^{b-1} b e^{\lambda\left(1-e^{x_i^{\beta}}\right)} \lambda e^{x_i^{\beta}} x_i \left(1-e^{\lambda\left(1-e^{x_i^{\beta}}\right)}\right)^{b} \log\left(1-e^{\lambda\left(1-e^{x_i^{\beta}}\right)}\right)}{1-\left(1-\left(1-e^{\lambda\left(1-e^{x_i^{\beta}}\right)}\right)^{b}\right)^{2}} \\
& - \sum_{i=1}^{n} \frac{2\left(1-e^{\lambda\left(1-e^{x_i^{\beta}}\right)}\right)^{b-1} b e^{\lambda\left(1-e^{x_i^{\beta}}\right)} \lambda e^{x_i^{\beta}} x_i \left(1-\left(1-e^{\lambda\left(1-e^{x_i^{\beta}}\right)}\right)^{b}\right)}{\left(1-\left(1-\left(1-e^{\lambda\left(1-e^{x_i^{\beta}}\right)}\right)^{b}\right)^{2}\right)^{2}} \\
& \times 2\left(1-e^{\lambda\left(1-e^{x_i^{\beta}}\right)}\right)^{b} \log\left(1-e^{\lambda\left(1-e^{x_i^{\beta}}\right)}\right) \left(1-\left(1-e^{\lambda\left(1-e^{x_i^{\beta}}\right)}\right)^{b}\right),
\end{aligned} \tag{8}
$$

$$
\begin{aligned}
\frac{\partial^2 \ell}{\partial \lambda^2} = &\sum_{i=1}^{n} \frac{e^{\lambda\left(1-e^{x_i^{\beta}}\right)^{b}} \left(\left(1-e^{x_i^{\beta}}\right)^{b}\right)^{2}}{1-\left(1-e^{\lambda\left(1-e^{x_i^{\beta}}\right)^{b}}\right)} \\
&-\frac{n}{\lambda^2}+\sum_{i=1}^{n}(b-1)\frac{e^{\lambda\left(1-e^{x_i^{\beta}}\right)}\left(1-e^{x_i^{\beta}}\right)^{2}}{1-e^{\lambda\left(1-e^{x_i^{\beta}}\right)}}+\sum_{i=1}^{n}\frac{\left(e^{\lambda\left(1-e^{x_i^{\beta}}\right)}\left(1-e^{x_i^{\beta}}\right)\right)^{2}}{\left(1-e^{\lambda\left(1-e^{x_i^{\beta}}\right)}\right)^{2}} \\
&-(\alpha-1)\sum_{i=1}^{n}\frac{2\left(1-e^{\lambda\left(1-e^{x_i^{\beta}}\right)}\right)^{b-1} b e^{\lambda\left(1-e^{x_i^{\beta}}\right)}\left(1-e^{x_i^{\beta}}\right)^{2}}{1-\left(1-\left(1-e^{\lambda\left(1-e^{x_i^{\beta}}\right)}\right)^{b}\right)^{2}} \\
&-\sum_{i=1}^{n}\frac{\left(1-e^{\lambda\left(1-e^{x_i^{\beta}}\right)}\right)^{b-2}(b-1)\, e^{\lambda\left(1-e^{x_i{}^{\beta}}\right)^{2}}\left(1-e^{x_i^{\beta}}\right)^{2} b\left(1-\left(1-e^{\lambda\left(1-e^{x_i^{\beta}}\right)}\right)^{b}\right)}{1-\left(1-\left(1-e^{\lambda\left(1-e^{x_i^{\beta}}\right)}\right)^{b}\right)^{2}} \\
&+\sum_{i=1}^{n}\frac{\left(\left(1-e^{\lambda\left(1-e^{x_i^{\beta}}\right)}\right)^{b-1} b e^{\lambda\left(1-e^{x_i^{\beta}}\right)}\left(1-e^{x_i^{\beta}}\right)\right)^{2}}{1-\left(1-\left(1-e^{\lambda\left(1-e^{x_i^{\beta}}\right)}\right)^{b}\right)^{2}} \\
&+\sum_{i=1}^{n}\frac{4\left(\left(1-e^{\lambda\left(1-e^{x_i^{\beta}}\right)}\right)^{b-1} b e^{\lambda\left(1-e^{x_i^{\beta}}\right)}\left(1-e^{x_i^{\beta}}\right)\left(1-\left(1-e^{\lambda\left(1-e^{x_i^{\beta}}\right)}\right)^{b}\right)\right)^{2}}{\left(1-\left(1-\left(1-e^{\lambda\left(1-e^{x_i^{\beta}}\right)}\right)^{b}\right)^{2}\right)^{2}},
\end{aligned}
\tag{9}
$$

$$
\begin{aligned}
\frac{\partial^2 \ell}{\partial \lambda \partial b} = & - \sum_{i=1}^{n} \frac{e^{\lambda\left(1-e^{x_i^{\beta}}\right)}\left(1-e^{x_i^{\beta}}\right)}{1-e^{\lambda\left(1-e^{x_i^{\beta}}\right)}} \\
& + \sum_{i=1}^{n} \frac{e^{\lambda\left(1-e^{x_i^{\beta}}\right)^{b}}\lambda\left(1-e^{x_i^{\beta}}\right)^{2b}\log\left(1-e^{x_i^{\beta}}\right)+e^{\lambda\left(1-e^{x_i^{\beta}}\right)^{b}}\left(1-e^{x_i^{\beta}}\right)^{b}\log\left(1-e^{x_i^{\beta}}\right)}{1-\left(1-e^{\lambda\left(1-e^{x_i^{\beta}}\right)^{b}}\right)} \\
& - \sum_{i=1}^{n} \frac{\left(e^{\lambda\left(1-e^{x_i^{\beta}}\right)^{b}}\right)^{2}\left(1-e^{x_i^{\beta}}\right)^{2b}\lambda\log\left(1-e^{x_i^{\beta}}\right)}{\left(1-\left(1-e^{\lambda\left(1-e^{x_i^{\beta}}\right)^{b}}\right)\right)^{2}} \\
& - (\alpha-1)\sum_{i=1}^{n} \frac{2\left(\left(1-e^{\lambda\left(1-e^{x_i^{\beta}}\right)}\right)^{b-1}\log\left(1-e^{\lambda\left(1-e^{x_i^{\beta}}\right)}\right)be^{\lambda\left(1-e^{x_i^{\beta}}\right)}\left(1-e^{x_i^{\beta}}\right)\right)}{1-\left(1-\left(1-e^{\lambda\left(1-e^{x_i^{\beta}}\right)}\right)^{b}\right)^{2}} \\
& + \sum_{i=1}^{n} \frac{\left(1-e^{\lambda\left(1-e^{x_i^{\beta}}\right)}\right)^{b-1}e^{\lambda\left(1-e^{x_i^{\beta}}\right)}\left(1-e^{x_i^{\beta}}\right)\left(1-\left(1-e^{\lambda\left(1-e^{x_i^{\beta}}\right)}\right)^{b}\right)}{1-\left(1-\left(1-e^{\lambda\left(1-e^{x_i^{\beta}}\right)}\right)^{b}\right)^{2}} \\
& - \sum_{i=1}^{n} \frac{b\left(1-e^{\lambda\left(1-e^{x_i^{\beta}}\right)}\right)^{2b-1}e^{\lambda\left(1-e^{x_i^{\beta}}\right)}\left(1-e^{x_i^{\beta}}\right)\log\left(1-e^{\lambda\left(1-e^{x_i^{\beta}}\right)}\right)}{1-\left(1-\left(1-e^{\lambda\left(1-e^{x_i^{\beta}}\right)}\right)^{b}\right)^{2}} \\
& - \sum_{i=1}^{n} \frac{2\left(1-e^{\lambda\left(1-e^{x_i^{\beta}}\right)}\right)^{b-1}be^{\lambda\left(1-e^{x_i^{\beta}}\right)}\left(1-e^{x_i^{\beta}}\right)\left(1-\left(1-e^{\lambda\left(1-e^{x_i^{\beta}}\right)}\right)^{b}\right)}{\left(1-\left(1-\left(1-e^{\lambda\left(1-e^{x_i^{\beta}}\right)}\right)^{b}\right)^{2}\right)^{2}} \\
& \times 2\left(1-e^{\lambda\left(1-e^{x_i^{\beta}}\right)}\right)^{b}\log\left(1-e^{\lambda\left(1-e^{x_i^{\beta}}\right)}\right)\left(1-\left(1-e^{\lambda\left(1-e^{x_i^{\beta}}\right)}\right)^{b}\right),
\end{aligned}
\tag{10}
$$

$$
\begin{aligned}
\frac{\partial^2 \ell}{\partial b^2} = & \sum_{i=1}^{n} \frac{e^{\lambda\left(1-e^{x_i^{\beta}}\right)^b}\left(\lambda\left(1-e^{x_i^{\beta}}\right)^b \log\left(1-e^{x_i^{\beta}}\right)\right)^2 + e^{\lambda\left(1-e^{x_i^{\beta}}\right)^b}\lambda\left(1-e^{x_i^{\beta}}\right)^b \log\left(1-e^{x_i^{\beta}}\right)^2}{1-\left(1-e^{\lambda\left(1-e^{x_i^{\beta}}\right)^b}\right)} \\
& - \sum_{i=1}^{n} \frac{\left(e^{\lambda\left(1-e^{x_i^{\beta}}\right)^b}\lambda\left(1-e^{x_i^{\beta}}\right)^b \log\left(1-e^{x_i^{\beta}}\right)\right)^2}{\left(1-\left(1-e^{\lambda\left(1-e^{x_i^{\beta}}\right)^b}\right)\right)^2} \\
& - \frac{n}{b^2} + (\alpha-1)\sum_{i=1}^{n} \frac{2\left(1-e^{\lambda\left(1-e^{x_i^{\beta}}\right)}\right)^b \log\left(1-e^{\lambda\left(1-e^{x_i^{\beta}}\right)}\right)^2\left(1-\left(1-e^{\lambda\left(1-e^{x_i^{\beta}}\right)}\right)^b\right)}{1-\left(1-\left(1-e^{\lambda\left(1-e^{x_i^{\beta}}\right)}\right)^b\right)^2} \\
& - \sum_{i=1}^{n} \frac{\left(\left(1-e^{\lambda\left(1-e^{x_i^{\beta}}\right)}\right)^b \log\left(1-e^{\lambda\left(1-e^{x_i^{\beta}}\right)}\right)\right)^2}{1-\left(1-\left(1-e^{\lambda\left(1-e^{x_i^{\beta}}\right)}\right)^b\right)^2} \\
& - \sum_{i=1}^{n} \frac{4\left(\left(1-e^{\lambda\left(1-e^{x_i^{\beta}}\right)}\right)^b \log\left(1-e^{\lambda\left(1-e^{x_i^{\beta}}\right)}\right)\left(1-\left(1-e^{\lambda\left(1-e^{x_i^{\beta}}\right)}\right)^b\right)\right)^2}{\left(1-\left(1-\left(1-e^{\lambda\left(1-e^{x_i^{\beta}}\right)}\right)^b\right)^2\right)^2}.
\end{aligned}
\tag{11}
$$
